# Cloacal microbiomes of sympatric and allopatric *Sceloporus* lizards vary with environment and host relatedness

**DOI:** 10.1371/journal.pone.0279288

**Published:** 2022-12-22

**Authors:** Marie E. Bunker, Stacey L. Weiss

**Affiliations:** University of Puget Sound, Tacoma, WA, United States of America; CNRS: BIOM Integrative Biology of Marine Organisms, FRANCE

## Abstract

Animals and their microbiomes exert reciprocal influence; the host’s environment, physiology, and phylogeny can impact the composition of the microbiome, while the microbes present can affect host behavior, health, and fitness. While some microbiomes are highly malleable, specialized microbiomes that provide important functions can be more robust to environmental perturbations. Recent evidence suggests *Sceloporus virgatus* has one such specialized microbiome, which functions to protect eggs from fungal pathogens during incubation. Here, we examine the cloacal microbiome of three different *Sceloporus* species (spiny lizards; Family Phrynosomatidae)–*Sceloporus virgatus*, *Sceloporus jarrovii*, and *Sceloporus occidentalis*. We compare two species with different reproductive modes (oviparous vs. viviparous) living in sympatry: *S*. *virgatus* and *S*. *jarrovii*. We compare sister species living in similar habitats (riparian oak-pine woodlands) but different latitudes: *S*. *virgatus* and *S*. *occidentalis*. And, we compare three populations of one species (*S*. *occidentalis*) living in different habitat types: beach, low elevation forest, and the riparian woodland. We found differences in beta diversity metrics between all three comparisons, although those differences were more extreme between animals in different environments, even though those populations were more closely related. Similarly, alpha diversity varied among the *S*. *occidentalis* populations and between *S*. *occidentalis* and *S*. *virgatus*, but not between sympatric *S*. *virgatus* and *S*. *jarrovii*. Despite these differences, all three species and all three populations of *S*. *occcidentalis* had the same dominant taxon, *Enterobacteriaceae*. The majority of the variation between groups was in low abundance taxa and at the ASV level; these taxa are responsive to habitat differences, geographic distance, and host relatedness. Uncovering what factors influence the composition of wild microbiomes is important to understanding the ecology and evolution of the host animals, and can lead to more detailed exploration of the function of particular microbes and the community as a whole.

## Introduction

The microbial community living within and on animal hosts has implications for host development, fitness, and evolution [1, 2]. While the influence of the microbiome on its host is well documented, the effect that host environment and phylogeny has on its microbial residents is less well understood, particularly in non-model and non-mammalian organisms that are free-ranging in their natural environment [3]. There is evidence that these communities are influenced by environmental factors, such as diet [4–7], geographic or elevation gradients [7–9], life stage [10, 11], or anthropogenic disturbance [12]. These factors can influence the “pool” of microbes available to colonize a host, as well as the internal environment of the host which impacts microbe establishment and propagation [8, 13, 14].

However, this malleability is not universal. Some microbiomes are closely tied to host evolution, and the genetics of that host is a critical factor impacting its microbial composition [3, 7, 14, 15]. These close host-microbiome relationships are often facilitated by vertical transmission from mother to offspring [5, 16, 17], and can be due to–or lead to–highly specialized microbiomes that perform a specific and vital function for the host. For example, *Euprymna scolopes* (Hawaiian bobtail squid) have a specialized organ to house a single species of bacteria which is integral to its fitness [18], and in several subspecies of Galapagos iguanas, which require specialized microbes to aid in digestion, genetic distance was found to be the most important factor for determining microbiome composition [15]. Because of the functional importance of the specific microbes, these communities are much more robust and less impacted by environmental changes. Emerging evidence suggests that *Sceloporus virgatus* (striped plateau lizards) possesses one such microbiome, which has undergone selection to favor microbes that protect eggs during incubation [19]. Specifically, the *S*. *virgatus* cloacal microbiome is largely dominated by members of *Enterobacteriaceae* [20, 21], which has been shown to have antifungal properties and likely protects eggs from fungal fouling during incubation [19, 22–24].

Here we study the effect of habitat and host genetics on the cloacal microbiome of three *Sceloporus* species from four different locations. With these data, we examine the differences between sympatric species, allopatric sister species, and intraspecific differences across three populations. We specifically compared microbiomes recovered from cloacal swabs, due to the specialized nature of *S*. *virgatus* cloacal microbes. First we looked at *S*. *virgatus* and *S*. *jarrovii*. These two species are sympatric in the Madrean Sky Islands of southwestern United States and northern Mexico, and have similar microhabitats, diets, and activity budgets, and so they are subject to the same environmental influences on their microbiomes [25–28]. However, *S*. *jarrovii* is a fairly distant relative of *S*. *virgatus*, is larger in body size, and is viviparous rather than oviparous. Next we compared *S*. *virgatus* to its oviparous sister species *S*. *occidentalis*, which is found throughout the western US [25, 29]. Despite being allopatric to *S*. *virgatus*, we chose a *S*. *occidentalis* population from a similar riparian oak-pine woodland habitat. Finally, we compared three populations of *S*. *occidentalis* from different habitats, which vary in primary substrates, elevation, and anthropogenic influence. Together, these comparisons can show the effect of both habitat and host relatedness on the cloacal microbiome. If habitat influences were acting alone on the microbiome, we would predict robust similarities in the microbiome of the more distantly related but sympatric species, and distinct differences in the microbiome of conspecifics in different habitats. In contrast, if host relatedness were acting alone on the microbiome, we would predict similarities across the three conspecific populations found in different habitats, and differences between the two sympatric species. Additionally, conservation of specific microbes across more closely related hosts could indicate vertical transmission of these microbes [16]. Strict vertical transmission is often an indicator that the conserved microbes serve an important function for the host (i.e., the antifungal properties of *Enterobacteriaceae* species).

## Methods

### Sample collection

**Fig 1:**
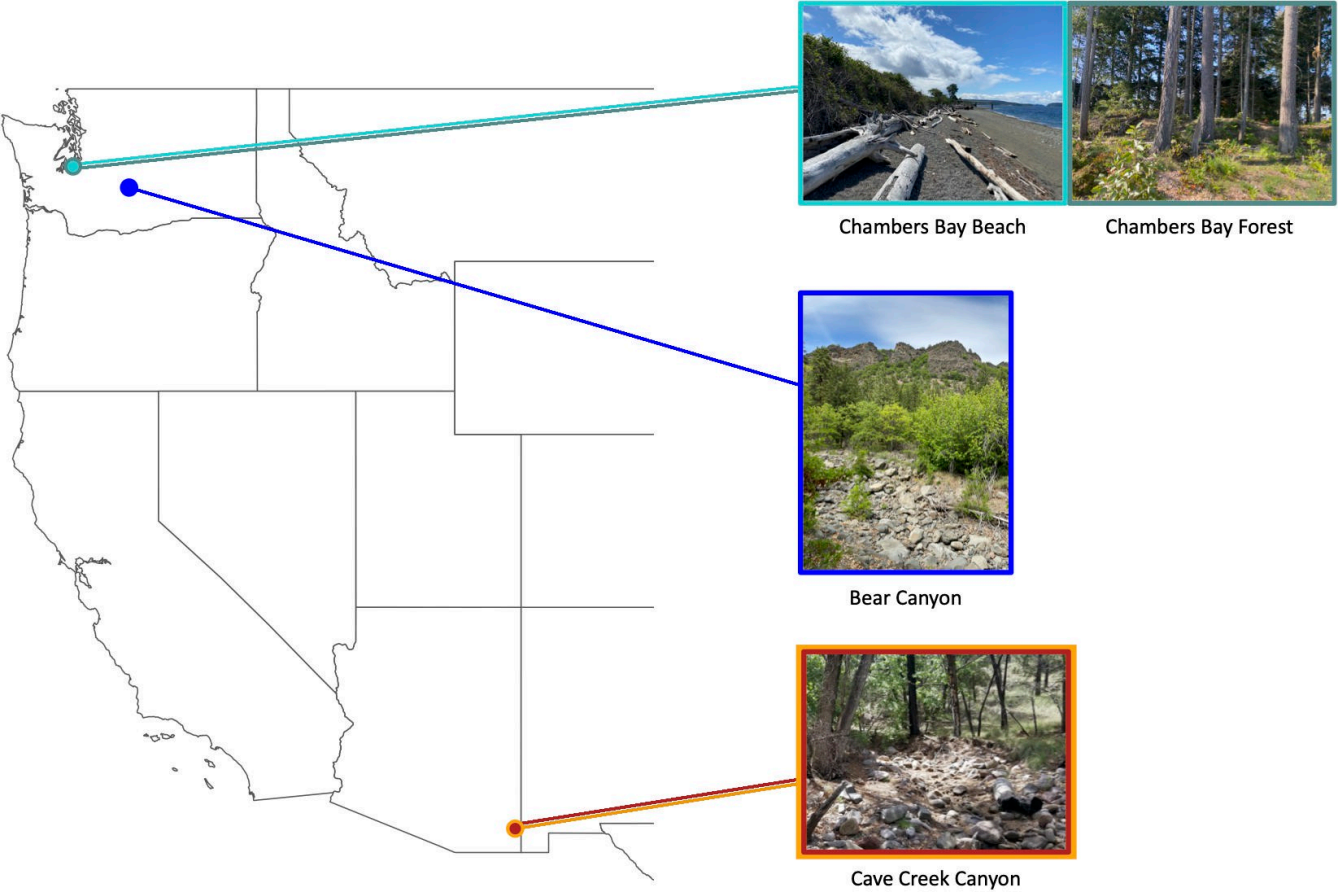
Study sites of *S*. *occidentalis* (Chambers Bay and Bear Canyon, Washington, USA), and *S*. *virgatus* and *S*. *jarrovii* (Cave Creek Canyon, Arizona, USA).

Female *Sceloporus virgatus* and *Sceloporus jarrovii* lizards were collected using a loop of fishing line at the end of a retractable fishing pole in the Coronado National Forest near the Southwestern Research Station (SWRS), Cochise County, AZ, from 17 July to 28 August 2017 and 16 July to 19 July 2021 (Fig 1). These sympatric lizards were collected along an intermittent creek surrounded by an oak-pine mixed forest in Cave Creek Canyon at ~1700 m a.s.l. They share microhabitat and can be found within a few body lengths of each other, though *S*. *virgatus* is more commonly found on small rocks whereas *S*. *jarrovii* is more commonly found on larger rock walls. Both male and female *Sceloporus occidentalis* were captured in Washington State from 29 July to 26 August 2020 and 7 July to 27 July 2021 at three locations that represent three distinct habitats: Beach, Forest, and Canyon (Fig 1). The Beach population (Chambers Creek Regional Park, University Place, WA) is at sea level (0 m elevation) on the coast of the Puget Sound; the habitat primarily consists of large pieces of driftwood with small clumps of beach grass and shrubs. The Forest population is located ~1.2 km from the Beach population within the same public park at ~70 m elevation; the habitat is a narrow (24 m) wooded strip of land sandwiched between a paved public walking trail and a road. The Canyon population (Oak Creek Wildlife Area, Yakima County, WA) is at ~640 m elevation; the study site follows an intermittent creek surrounded by an oak-pine forest, resemblant of the Cave Creek Canyon habitat. Only the Canyon *S*. *occidentalis* population was directly compared to *S*. *virgatus* due to the similar riparian oak-pine habitat.

All lizard cloacae (Table 1) were swabbed in the field immediately upon capture by gently inserting a sterile swab (BD ESwab™) into the cloaca and slowly rotating it. Swabs were kept in Amies solution on ice packs and/or refrigerated for up to 30 h until microbial samples could be moved to -80°C for later DNA extraction. Animals were released at the location of capture after swabbing and collection of demographic information (body mass, snout-vent length, and sex). Swabbing methods are minimally invasive, and animals behaved normally immediately upon release. All lizards were adults and sampling occurred after the conclusion of the reproductive season.

**Table 1:** Sample sizes of swabs collected from *Sceloporus* lizards. NA indicates that we did not collect swabs from male *S*. *virgatus* and *S*. *jarrovii* lizards. “After Processing” is the number of swabs that were retained after removing fecal-contaminated swabs. See Methods for details.

	Total		After Processing	
Group	Female	Male	Female	Male
*S*. *virgatus*	19	NA	13	NA
*S*. *jarrovii*	24	NA	18	NA
*S*. *occidentalis* (Canyon)	21	25	14	9
*S*. *occidentalis* (Forest)	22	24	15	19
*S*. *occidentalis* (Beach)	26	24	21	16

Our work was approved by the Institutional Animal Care and Use Committee of the University of Puget Sound (Protocol Number: PS16002 and PS18002). All animal collections were permitted by Arizona Game and Fish (SP590934 and SP846957) and Washington Fish and Wildlife (20–039 and 21–002).

### DNA extraction and Illumina library

All samples were extracted with the Qiagen DNEasyⓇ Blood and Tissue Kit (Qiagen, Inc), using the manufacturer protocol for Purification of Total DNA from Animal Blood or Cells, with the optional pre-treatment for gram-positive bacteria lysis buffer incubation. An extraction blank was included with each set of extractions. DNA in all samples and blanks was quantified via Qubit, to ensure extraction was successful. Briefly, Illumina libraries were prepared via a two-step polymerase chain reaction (PCR). PCR1 utilized 515F/806R primer pairs to amplify the V4 region of the 16s rRNA bacterial gene. PCR2 added unique barcode primers to each sample’s sequences. Samples were then pooled according to qualitative DNA concentration and sent to the University of Idaho Genomics and Bioinformatics Core (GRBC) for sequencing on the Illumina MiSeq platform. Details found in Bunker et al., 2021a, b [19, 20].

### Sample processing

Sequences were received demultiplexed, with adapters and primers removed. Quality analysis for each sample was performed using FastQC [30] and those results were consolidated using MultiQC [31]. Prior to processing, samples with fecal contamination were removed based on the relative abundance of *Lachnospiraceae*, a fecal indicator species [20, 21]

Mean quality scores and length distribution for the whole dataset was manually inspected and used to determine a cutoff length of 250 bp for forward reads and 170 bp for reverse reads. Samples were then processed in R v 4.1.2 using the DADA2 [32] pipeline, based on this tutorial: https://benjjneb.github.io/dada2/tutorial.html. Samples were trimmed and filtered with a max expected error of 2. Taxonomic classification of amplified sequence variants (ASVs) was performed through the assignTaxonomy function, using the Silva database [33], release 132. Potential contaminants were removed with the Decontam package [34], using the “prevalence” method with a threshold of 0.1. Control samples (n = 57), including experimental controls, extraction blanks, and PCR negatives, were used for comparison. Any ASV that had fewer than 15 reads across all samples was discarded. Finally, read numbers were log transformed to account for differences in read depth. All parameters were determined based on an analysis of the mock communities (S1 Fig).

### Data analysis

Once samples had been processed, the phyloseq package [35] was used to organize and store data of different types for analysis. Shannon diversity index values and richness were calculated with the “estimate_richness” function from phyloseq, and Faith’s phylogenetic diversity index values (PD) were calculated using the Picante package [36] based on maximum-likelihood phylogenetic trees created and optimized with Phangorn, using the GTR substitution model [37, 38]. The alignment was created with the DECIPHER package [39]. Significance was defined as a p-value of < 0.05 for all the following analyses.

We tested for among-species differences in (log-transformed) alpha diversity metrics using one-way ANOVA, after confirming the log-transformed data met assumptions of normality and equal variance based on standard diagnostic plots of model residuals. ANOVA’s were followed by two post hoc Dunnet’s tests comparing the sympatric species (*S*. *virgatus* vs. *S*. *jarrovii*) and the sister species (*S*. *virgatus* vs. *S*. *occidentalis*); only Canyon *S*. *occidentalis* were used in these analyses to match habitat type. Beta diversity and composition were compared among species using permutational-ANOVA (“adonis” in the vegan package; [40] using Bray-Curtis, weighted UniFrac, and unweighted UniFrac distances. Variance of the communities was also measured for all three metrics using “betadisper”, also from the vegan package [40].

We tested for among-population differences in alpha and beta diversity of the three *S*. *occidentalis* populations (Canyon, Forest, and Beach) in a similar way, but these analyses also included sex and a sex X location interaction, and were followed by Tukey post hoc tests to assess all pairwise comparisons. Richness and PD were log transformed.

The corncob package [41] was used for differential abundance analyses. The differentialTest command was first used to identify which ASVs varied between groups, and the model for each of these ASVs was specifically tested using the bbdml function. Shared taxa between groups were identified with the function “get_vennlist” from the Microbiota Processes package [42]. Data points for beta diversity plots were generated using the “ordinate” function from the Phyloseq package [35], and all plots were made using ggplot2 [43]. A dendrogram was created with the Bray-Curtis distance of mean communities, using hclust (base R) and the complete linkage method.

## Results

### Species in sympatry

**Fig 2:**
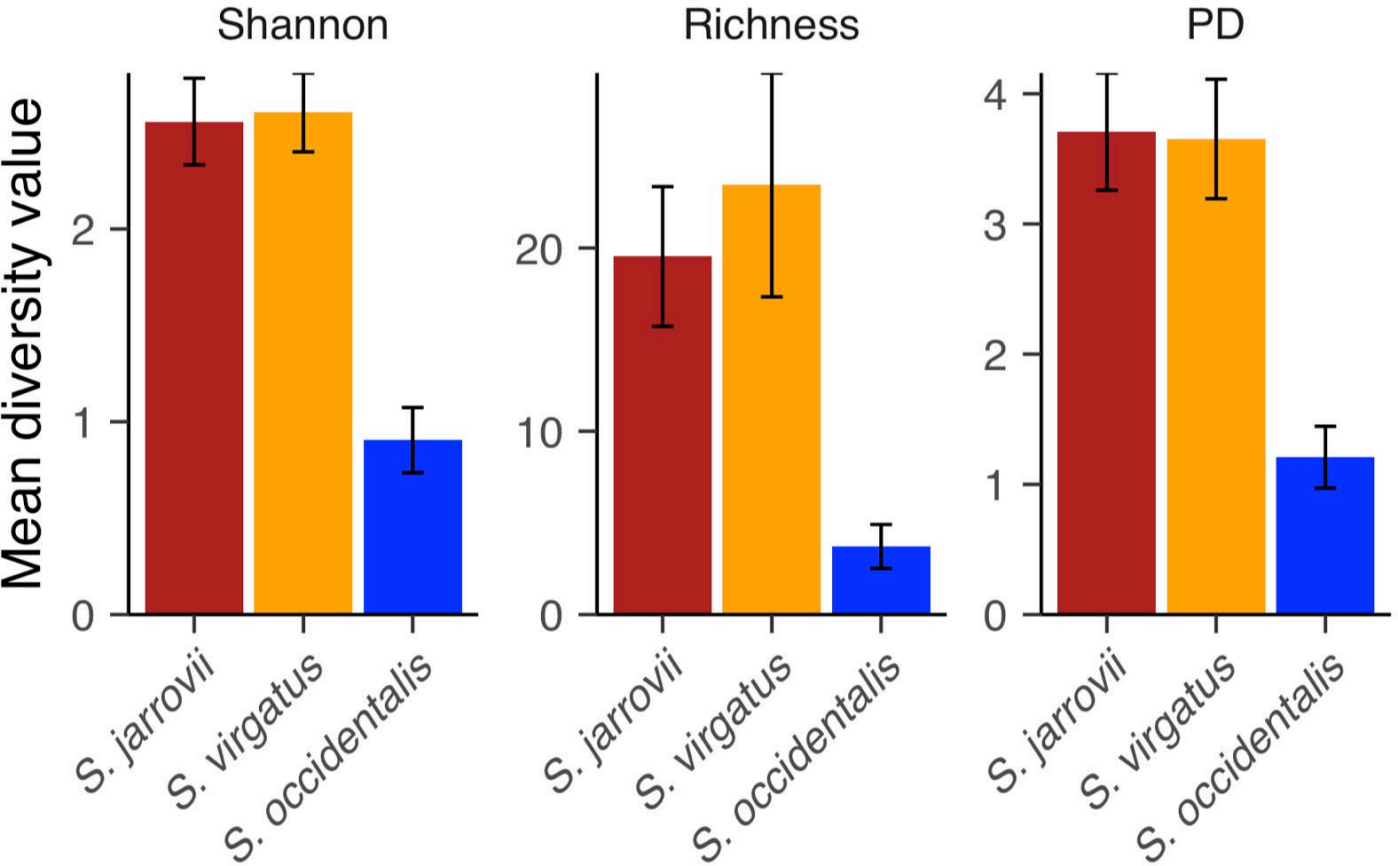
Mean Shannon diversity, richness, and PD from female *S*. *virgatus*, *S*. *jarrovii*, *S*. *occidentalis* (Canyon population) microbial communities recovered from cloacal swabs. Error bars represent standard error.

**Fig 3:**
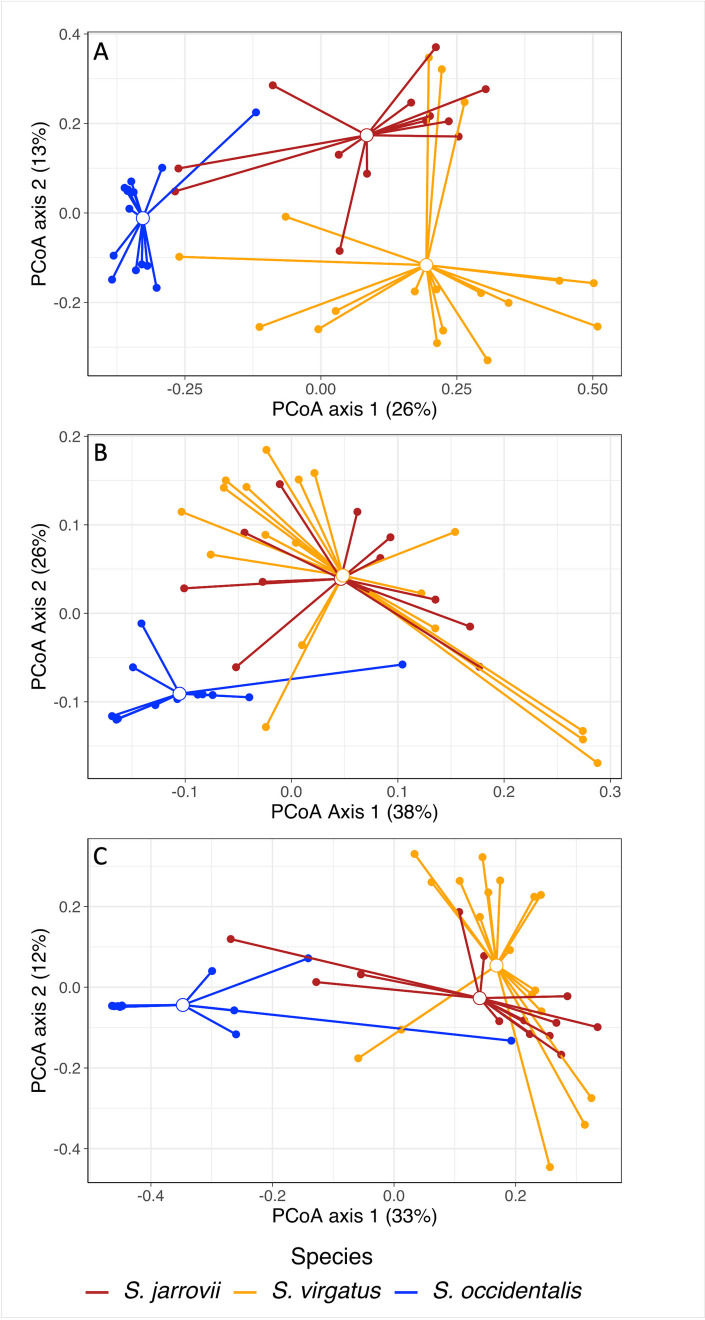
Principal coordinate analysis plots (PCoA) plot of microbial communities recovered from female *S*. *virgatus*, *S*. *jarrovii*, and *S*. *occidentalis* (Canyon population) cloacal swabs. Plots were created by using (A) Bray Curtis, (B) weighted Unifrac and (C) unweighted UniFrac distance to calculate pairwise distances based on community composition. Endpoints represent individual samples, connected to the centroid for each species. Colors represent species.

We compared the cloacal microbiomes of female *S*. *virgatus* and *S*. *jarrovii*, two species that live sympatrically in Cave Creek Canyon, with overlap in microhabitat choice. There was no difference between alpha diversity of the two species in any of the three metrics (Fig 2 and Table A in S1 File). However, the overall community varied in composition for all three beta diversity metrics (Fig 3A–3C and Table B in S1 File). Dispersion was similar across groups (Table B in S1 File).

**Fig 4:**
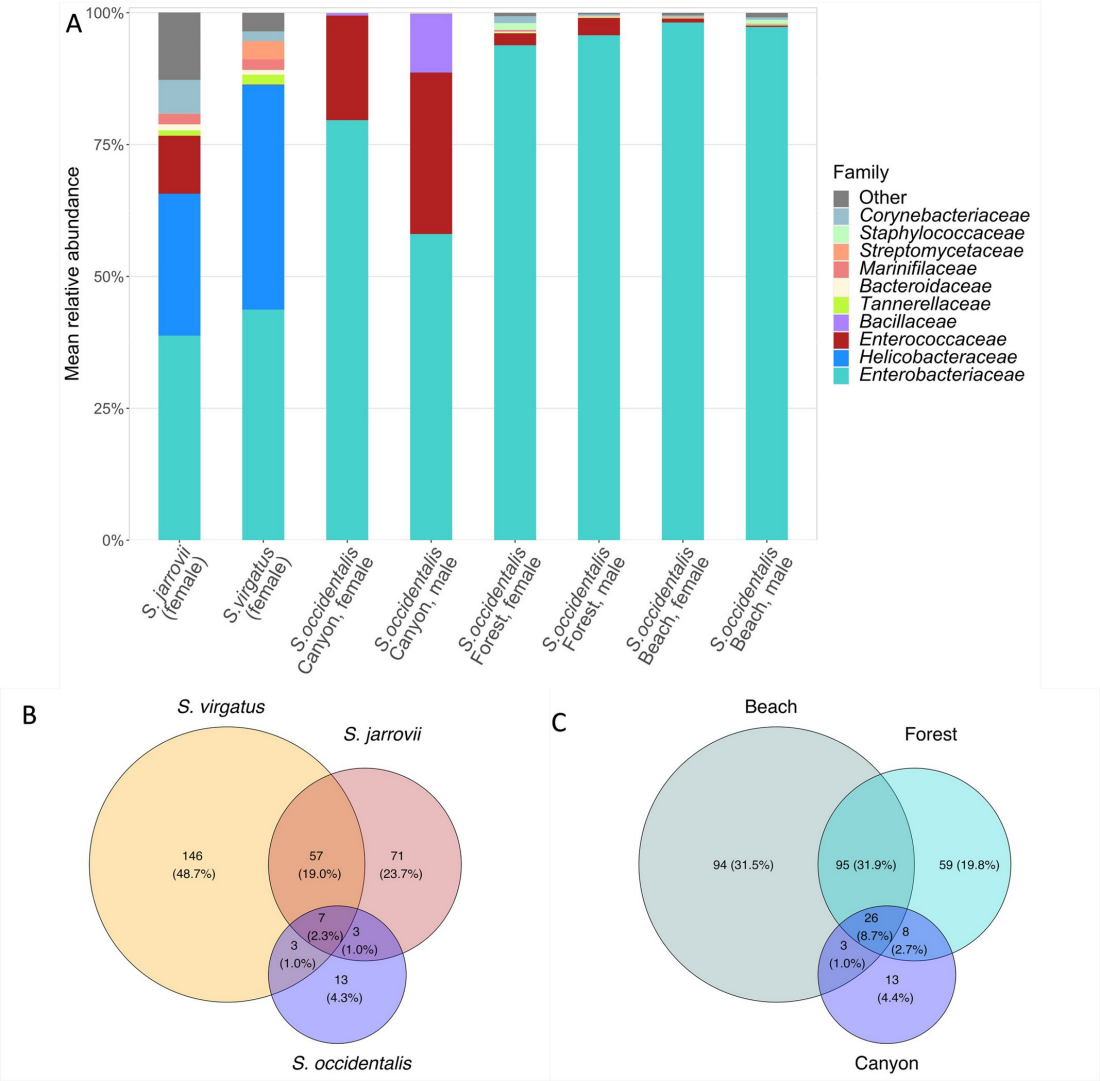
Composition of microbial communities recovered from female S. *virgatus*, *S*. *jarrovii*, and *S*. *occidentalis* (Canyon population) cloacal swabs. (A) Percent composition of the top 10 most abundant bacterial families from *S*. *jarrovii*, *S*. *virgatus*, and three *S*. *occidentalis* populations (remaining families were grouped into the “Other” category). Colored portions of the bars represent the mean relative abundance of each family, represented by the y-axis. Each bar represents the mean community. (B) Number and percent of shared ASVs between S. *virgatus*, *S*. *jarrovii*, and *S*. *occidentalis* (Canyon population) females. (C) Number and percent of shared ASVs between three *S*. *occidentalis* populations: Canyon, Forest, and Beach.

**Fig 5:**
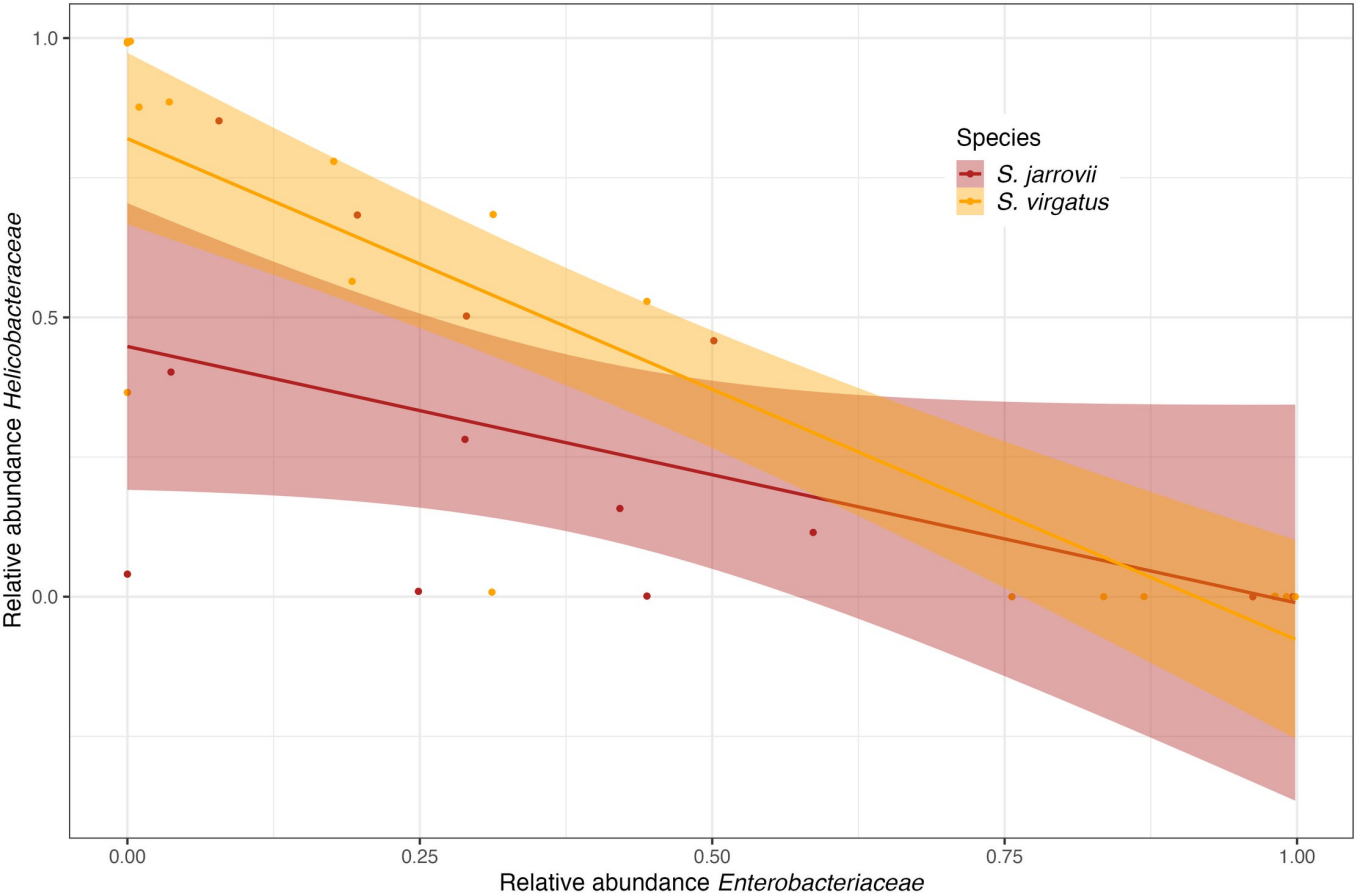
Pearson’s correlation of the relative abundances of the *Enterobacteriaceae* and *Helicobacteraceae* families in *S*. *virgatus* and *S*. *jarrovii* cloacal communities. Relative abundances of the two families were significantly correlated in *S*. *virgatus* (r = -0.88, df = 16, p < 0.001) but not *S*. *jarrovii* (r = -0.51, df = 11, p = 0.079).

*Enterobacteriaceae* and *Helicobacteraceae* are the most abundant families in both *S*. *virgatus* (43.8% ± 9.7 and 42.6% ± 9.9) and *S*. *jarrovii* (38.8% ± 8.8 and 26.9% ± 8.0, Fig 4A and S2 File). We observed that the relative abundance of *Enterobacteriaceae* appeared to increase with a decrease in relative abundance of *Helicobacteraceae*; a Pearson’s correlation confirmed this relationship to be significant (Fig 5). The two families together made up 86.4% ± 5.0, on average. This relationship was not significant in *S*. *jarrovii*. No other families made up >2% of the *S*. *virgatus* community on average, whereas the *S*. *jarrovii* microbial community was, on average, made up of 11.0% ± 4.1 *Enterococcaceae* and 6.4% ± 2.8 *Corynebacteriaceae*. Overall, 87% ± 4.8 of the community of *S*. *virgatus* are members of the Proteobacteria phylum, with the remainder of the community closely split between Actinobacteria (5.7% ± 3.5), Bacteroidetes (5.5% ± 3.4), and Firmicutes (1.0% ± 3.9) S2 File). The *S*. *jarrovii* community is also majority Proteobacteria at an average of 68.9 ± 7.2%, but has a greater relative abundance of Firmicutes (15.6% ± 4.8), and a similar amount of Actinobacteria (7.2% ± 2.9) and Bacteroidetes (5.6% ± 3.3). No individual ASV was found to be significantly differentially abundant between species. *S*. *virgatus* and *S*. *jarrovii* shared 21.5% of ASVs, with 48.3% unique to *S*. *virgatus*, and 23.5% unique to *S*. *jarrovii* (Fig 4B).

### Species in allopatry

We compared cloacal microbiomes of *S*. *virgatus* and Canyon *S*. *occidentalis*, sister species from different latitudes but both in oak-pine woodlands with similar microhabitat selection. We found that *S*. *virgatus* had significantly higher diversity than *S*. *occidentalis* in all three alpha metrics (Fig 2 and Table A in S1 File). The composition of the bacterial communities also varied in both dispersion and composition (Fig 3A–3C and Table B in S1 File).

Like the *S*. *virgatus* communities (described above), the *S*. *occidentalis* bacterial populations were largely dominated by *Enterobacteriaceae*, which made up 79.6% ± 6.8 of the community on average (Fig 4A and S2 File). Unlike *S*. *virgatus* and *S*. *jarrovii*, there was only one *S*. *occidentalis* individual with any amount of *Helicobacteraceae*, and that amount was negligible (< 0.1% of the community). Instead, the next most abundant family was *Enterococcaceae* at 19.8% ± 6.9 relative abundance on average, but this family was present only in small abundances in the *S*. *virgatus* samples. No other family made up > 1% of the *S*. *occidentalis* community on average. This pattern was mirrored at the Phylum level, with 79.7% ± 6.8 Proteobacteria and 20.3% ± 6.8 Firmicutes, with no other major taxa (S2 File). A differential abundance analysis confirms that *Helicobacteraceae* is significantly higher in *S*. *virgatus* (t = -8.63, p < 0.001). One *Enterobacteriaceae* ASV, genus *Salmonella*, was significantly less abundant in *S*. *virgatus* (t = -3.94, p = 0.002). Of the total ASVs, 4.6% were unique to *S*. *occidentalis*, and only 3.3% were shared between *S*. *occidentalis* and *S*. *virgatus* (Fig 4B).

### Intraspecies population comparison

**Fig 6:**
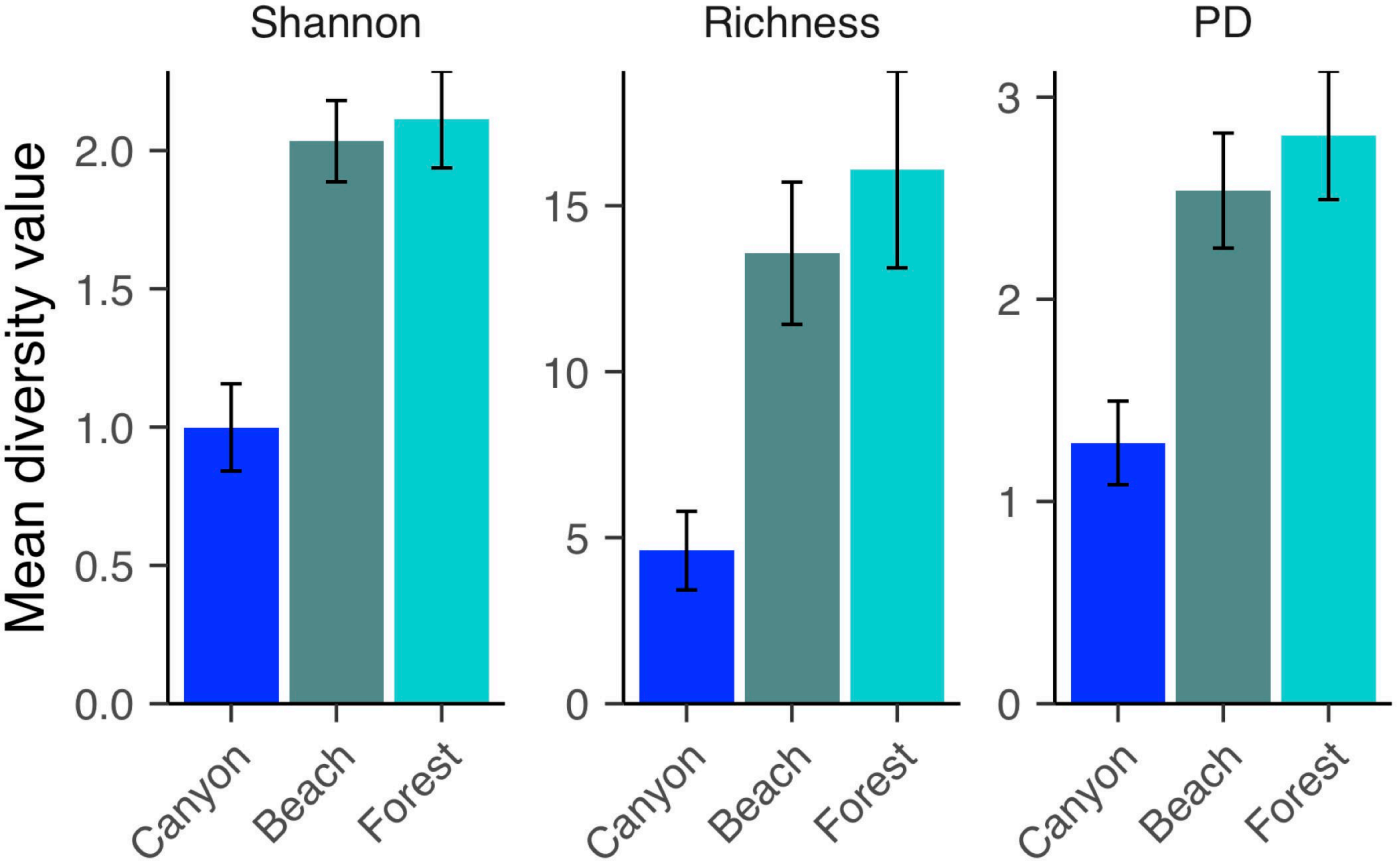
Mean Shannon diversity, richness, and PD of microbial communities recovered from *S*. *occidentalis* cloacal swabs from three different populations: Canyon, Forest, and Beach. Error bars represent standard error.

**Fig 7:**
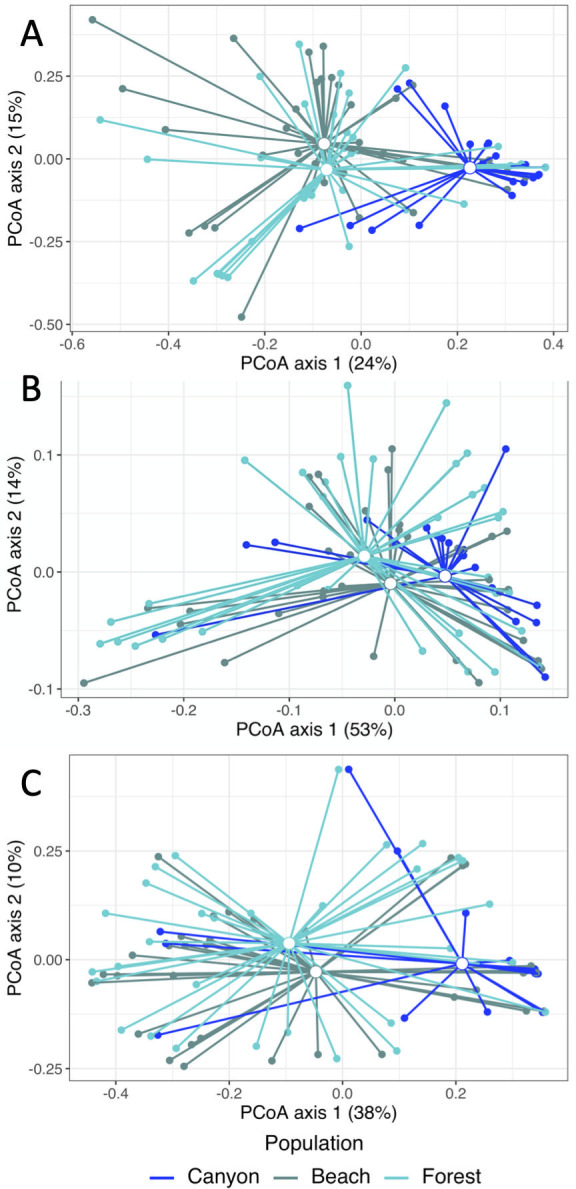
Principal coordinate analysis plots (PCoA) plot of cloacal microbial communities recovered from three *S*. *occidentalis* populations: Canyon, Forest, and Beach. Plots were created by using (A) Bray Curtis, (B) weighted Unifrac and (C) unweighted UniFrac distance to calculate pairwise distances based on community composition. Endpoints represent individual samples, connected to the centroid for each species. Colors represent populations.

We next compared different populations of *S*. *occidentalis* from three different habitats, in both males and females. We found no difference between the sexes, nor an interaction between sex and location in any of the alpha diversity metrics (Table C in S1 File). Location had an effect on all three metrics, with Canyon animals having significantly lower diversity than did the other two populations, which did not differ from each other (Fig 6 and Table C in S1 File). The microbial populations also varied in composition for all three metrics (Fig 7A–7C and Table D in S1 File), and in dispersion when using Bray-Curtis and unweighted UniFrac distances. Sexes did not differ in dispersion or composition of microbiome communities, although there was an interaction effect of sex and location only when using Bray-Curtis distance (Table D in S1 File).

**Fig 8:**
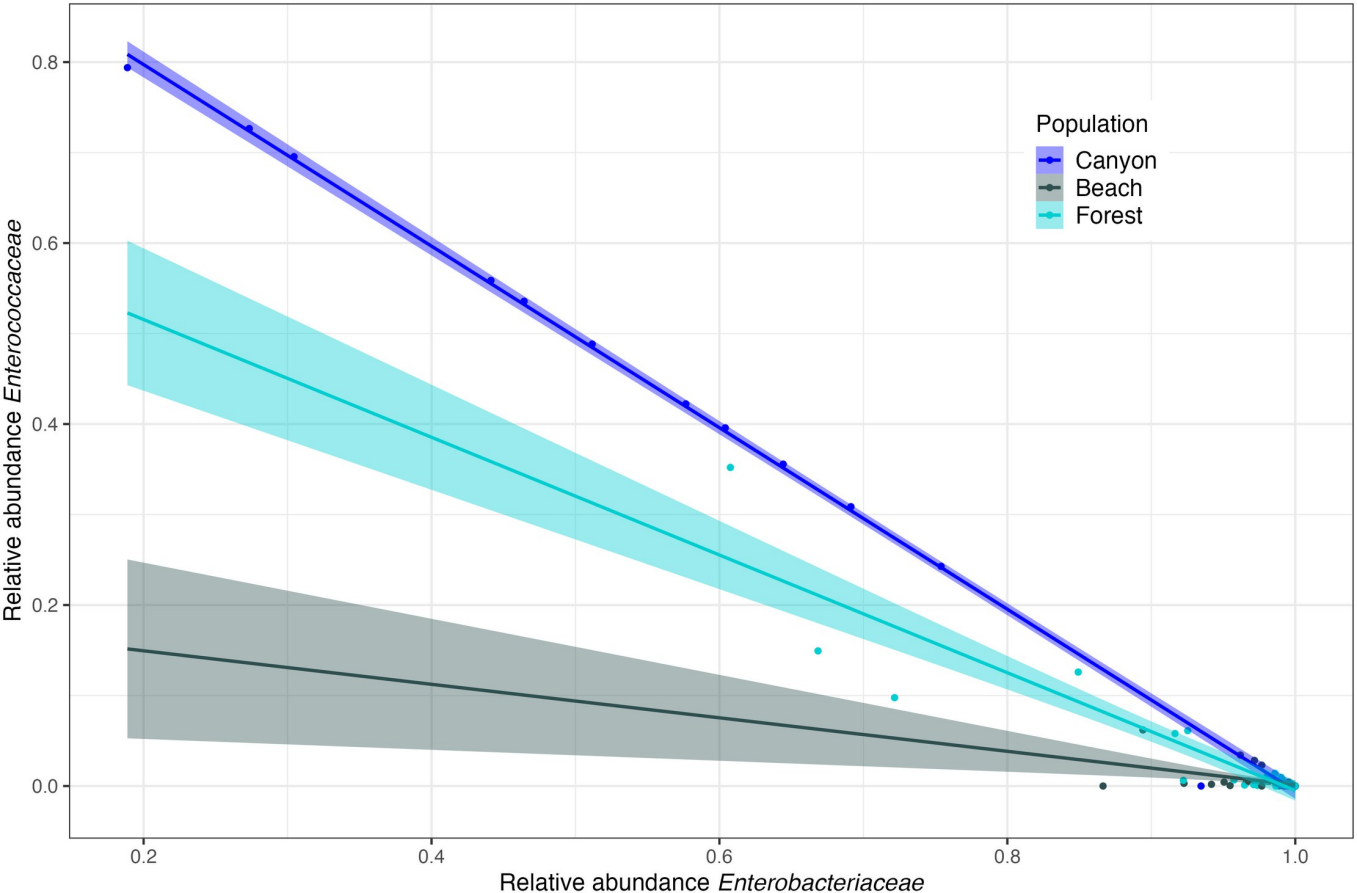
Pearson’s correlation of the relative abundances of the *Enterobacteriaceae* and *Enterococcaceae* families in *S*. *occidentalis* cloacal communities. The relationship was significant in all three populations (Canyon: r = 0.999, df = 20, p < 0.001; Beach: r = -0.45, df = 35, p = 0.005; Forest: r = -0.92, df = 31, p < 0.00), but was mostly closely correlated in the Canyon population.

The most abundant family in all three populations (for both males and females) was *Enterobacteriaceae* (Canyon: 71.2% ± 6.6, Forest: 94.9% ± 1.7, Beach: 97.8% ± 0.5, Fig 4A and S2 File). The Canyon and the Forest animals also hosted 24.0% ± 6.0 and 2.8% ± 1.2 *Enterococcaceae*, respectively, but no other taxa made up greater than 1% relative abundance on average in any population. *Enterococcaceae* showed a similar correlation with *Enterobacteriaceae* as did *Helicobacteriaceae* in the *S*. *virgatus* samples (Fig 8). The correlative relationship was present in all three populations, but relative abundances were most closely associated in the Canyon population. At the phyla level, Canyon populations were 71.2% ± 6.6 Proteobacteria and 28.7% ± 6.6 Firmicutes; Forest populations were 95.0% ± 1.6 Proteobacteria and 3.6% ± 1.5 Firmicutes; and Beach populations were 98.0% ± 0.5 Proteobacteria and 1.2% ± 0.4 Firmicutes (S2 File). This indicates that the major differences in diversity between these populations are in low abundance taxa, or at the ASV level. A differential abundance analysis confirms that *Enterococcaceae* is lower in both the Beach (t = -6.43, p < 0.001) and Forest (t = -3.44, p = 0.001) populations compared to the Canyon population. At the ASV level, 9.3% of sequences were shared between all three locations. The Beach and Forest populations (both at Chambers Creek Regional Park and separated by only ~1.2 km) shared 41.0% of their ASVs, while the Canyon population only shared 10.3 and 12.0% with the Beach and Forest populations, respectively (Fig 4C).

Of the 28 ASVs shared between all three locations (Fig 4C), five belonged to the *Lachnospiraceae* family (including one from the genus *Tyzzerella* and four that are unidentified at the genus level), four to family *Bacteroidaceae* (all from the *Bacteroides* genus), and three to both *Enterobacteriaceae* (*Salmonella*, *Citrobacter* and *Hafnia-Obesumbacterium* genera) and *Marinifilaceae* (all belonging to the *Odoribacter* genus) (S3 File).

### Dendrograms

**Fig 9:**
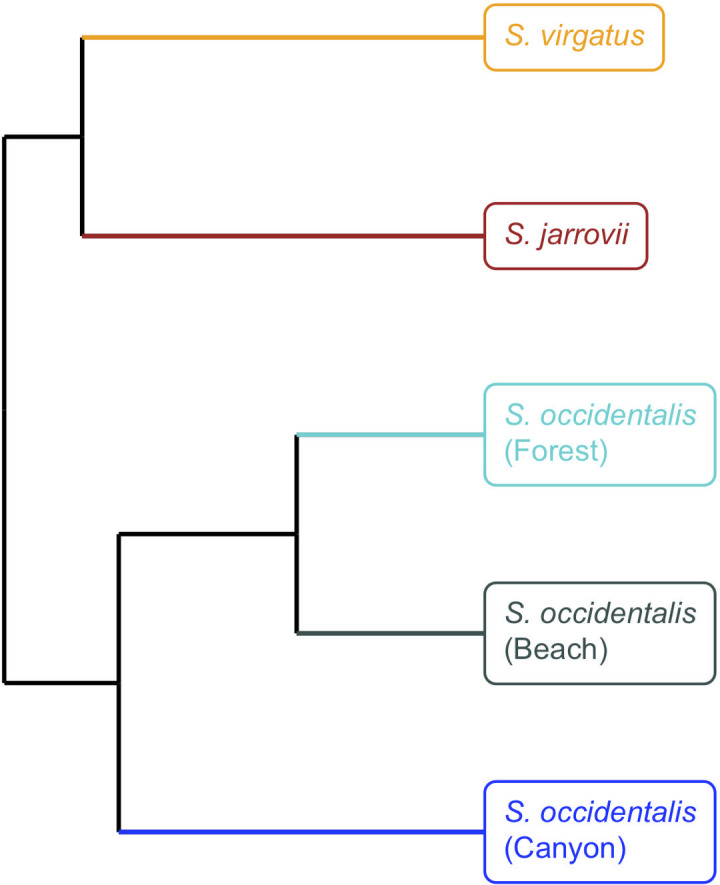
Dendrogram based on Bray-Curtis distance of mean clocal bacterial communities recovered from five *Sceloporus* populations: *S*. *virgatus*, *S*. *jarrovii*, and three populations of *S*. *occidentalis*.

We generated dendrograms to determine if clustering patterns of recovered microbial communities followed either of the patterns of interest in our study–species relatedness or habitat similarity. When mean communities of all five populations (*S*. *virgatus*, *S*. *jarrovii*, and three *S*. *occidentalis* populations) were clustered in dendrograms based on Bray-Curtis distance, samples grouped primarily by geographic distance (Fig 9). *S*. *virgatu*s clustered with *S*. *jarrovii* despite being more closely related to *S*. *occidentali*s, and the most geographically distant *S*. *occidentalis* population clustered separately from the two geographically close populations. Seven ASVs were shared among all three *Sceloporus* species (Fig 4B), all belonging to different genera: *Bacteroides*, *Bilophila*, *Enterococcus*, *Hafnia-Obesumbacterium*, *Helicobacter*, *Salmonella*, and an unknown *Veillonellaceae*.

## Discussion

We found that both environmental influences and host relatedness have an effect on the cloacal microbiome of three *Sceloporus* lizard species. The cloacal microbes of *S*. *occidentalis* animals from three different populations and three different habitat types were found to vary in alpha diversity, community composition, and community variance. This difference was driven by the Canyon population, which is geographically distinct from the Forest and Beach populations, which are only ~1.2 km apart from each other. The Canyon population of *S*. *occidentalis* is also distinct from its allopatric, oviparous sister species, *S*. *virgatus*, although they are found in similar habitats and thus may have similar environmental pressures. Finally, *S*. *virgatus* and *S*. *jarrovii*, more distantly related species that share a habitat, had similar alpha diversity metrics, and both species’ microbiomes are dominated by members of the *Enterobacteriaceae* and *Helicobacteraceae* families. However, *S*. *jarrovii* lacks the paired relationship of these two families that we see in *S*. *virgatus*, and also hosts a substantial amount of *Enterococcaceae*, which is essentially absent in *S*. *virgatus*. These two sympatric lizards have microbiomes that vary in beta diversity.

There are several environmental factors that may account for the differences in the *S*. *occidentalis* populations. Elevation changes have been known to induce changes in the microbiome, possibly due to an increase in stress as elevation increases [8, 44, 45]. There is also a much greater anthropogenic influence at the Beach and Forest populations, which are located in an urban recreational area, compared to the much more remote Canyon population. Human disturbance has been shown to influence the microbiome in a variety of species [8, 12, 46, 47]. Both of these factors have shown to influence the available reservoirs of microbes which can colonize a host, perhaps accounting for differences in microbiome composition as well as overall diversity metrics, including the lower diversity observed in the Canyon populations. We did not find consistent differences between the microbiomes of *S*. *occidentalis* males and females, which differs from other research on *S*. *virgatus* that found consistent differences between the sexes [21]. A larger sample size, or samples from time points during the reproductive season should be examined for *S*. *occidentalis* before a lack of sex difference is generalized for the species [48, 49]. Surely reproductive behaviors and physiology could also be drivers of microbial diversity [50, 51], but we would not capture that relationship here given our study was conducted at the conclusion of the annual reproductive season.

Environmental factors cannot account for all the differences seen here, as the community structure of *S*. *virgatus* and *S*. *jarrovii* microbiomes also differed, despite sharing a habitat. Variation between reptile species’ microbiomes has been hypothesized to be due to behavioral [50] or diet differences [5, 52]. However, microbes can also be influenced by host physiological and genetic factors. For instance, host genetic distance has been shown to predict variation in microbiome composition [14, 53–55]. This could be due to a selection process that winnows microbes that are picked up from the environments to a specific cohort of microbes [13, 14, 18]. Alternatively, portions of the microbiome could be vertically transmitted, and the differences due to inheritance. Vertical transmission is well established in viviparous animals, particularly mammals [17], and recent evidence suggests that oviparous animals can also pass down microbes to their offspring through behavioral transmission mechanisms or during egg development and oviposition [5, 56, 57]. The higher abundance of *Enterobacteriaceae* in *S*. *occidentalis* and *S*. *virgatus* compared to *S*. *jarrovii* could also be tied to mode of reproduction; there is evidence that the *S*. *virgatus* microbiome has been selected to protect eggs from fungal infection [19], a known property of some members of the *Enterobacteriaceae* family (most often *Serratia*) [22–24]. This selective pressure would be lacking from *S*. *jarrovii*, a viviparous species. Comparison of mother-daughter microbiomes, as well as quantifying the genetic distance between *Sceloporus* species could help determine what is causing these variations.

At a finer level, species in sympatry shared 21.5% of their ASV’s and conspecific populations ~1.2 km apart shared 41.0% of ASV’s, whereas more geographically distant populations of sister species and of conspecifics shared only 10–12% of ASV’s. This suggests that location determines the potential “pool” of microbes and, if that is similar, relatedness affects the likelihood of which microbes from the pool establish residency within the host. That environmental factors have the larger influence than relatedness is also supported by the fact that the microbiome of sympatric but distantly related species were more similar to each other than were those of allopatric sister species. However, the consistently high abundance of three major families (*Enterobacteriaceae*, *Helicobacteraceae*, and *Enterococcaceae*) across all species and habitats indicates strong conservation of the Family-level taxonomic profile and suggests some selection for the ecological function of these particular microbes.

Sampling of other reptile species, particularly with cloacal swabs, have also found that members of the Proteobacteria phyla dominate the cloacal microbiome, although it is less common to find such a high relative abundance of a single family as we do here. Colston [58] found that nearly 50% of microbes in the cloacae of the cottonmouth snake were Proteobacteria, with over 16% belonging to the Enterobacteriaceae family (although the single most abundant OTU was a *Bacteroidaceae* species). Helicobacteraceae has been shown to dominate the cloacal microbiomes of crocodile lizards [59], although it was impacted by the diet and health of the populations. Baldo et al. [53] found that Proteobacteria was the most diverse phyla in the gut tissue of several populations of wall lizards, and *Helicobacteraceae*, *Desulfovibrionaceae* and *Enterobacteriaceae* were found across all populations. Kohl et al. [5] found that female lizards of several viviparous species shared certain taxa with their offspring, and the majority of these shared taxa were members of the *Enterobacteriaceae* family, although the study used fecal samples rather than cloacal swabs.

Along with these previous findings, our results offer a starting point for further investigation into the proximate and ultimate mechanisms by which wild microbiomes are established within their hosts. More work should consider the relative role of vertical transmission vs. social/environmental/diet-based acquisition, how these processes are limited by host genetics, and the selective value of the host microbiome.

## Supporting information

S1 Fig Results of mock community analyses, showing the observed and expected mock community data. Three technical replicates of a tiered mock community were included in the three different Illumina runs that included samples analyzed in this manuscript.
(PDF)

S1 File Statistical output. Analyses of alpha and beta diversity metrics for the cloacal microbiome of *Sceloporus* lizards.
(PDF)

S2 File Relative abundances of the top 10 most abundant families and phyla in the cloacal microbiome of *Sceloporus* lizards. (PDF)

S3 File Identities of the 28 ASV’s shared among all 3 *Sceloporus occidentalis* populations. (PDF)
