# Supplementary material for: Cloacal microbiomes of sympatric and allopatric Sceloporus lizards vary with environment and host relatedness
Source: PLoS One. 2022 Dec 22;17(12):e0279288. doi: 10.1371/journal.pone.0279288 (PMC9779040; doi:10.1371/journal.pone.0279288)
Supplement: S1 Fig — Three technical replicates of a tiered mock community were included in the three different Illumina runs that included samples analyzed in this manuscript. (PDF) [file pone.0279288.s001.pdf]

M. E. Bunker and S. L. Weiss

Cloacal microbiomes of sympatric and allopatric *Sceloporus* lizards vary with environment and host relatedness

Supporting Information: S1 Figure. Results of mock community analyses, showing the observed and expected mock community data.

PLOS ONE

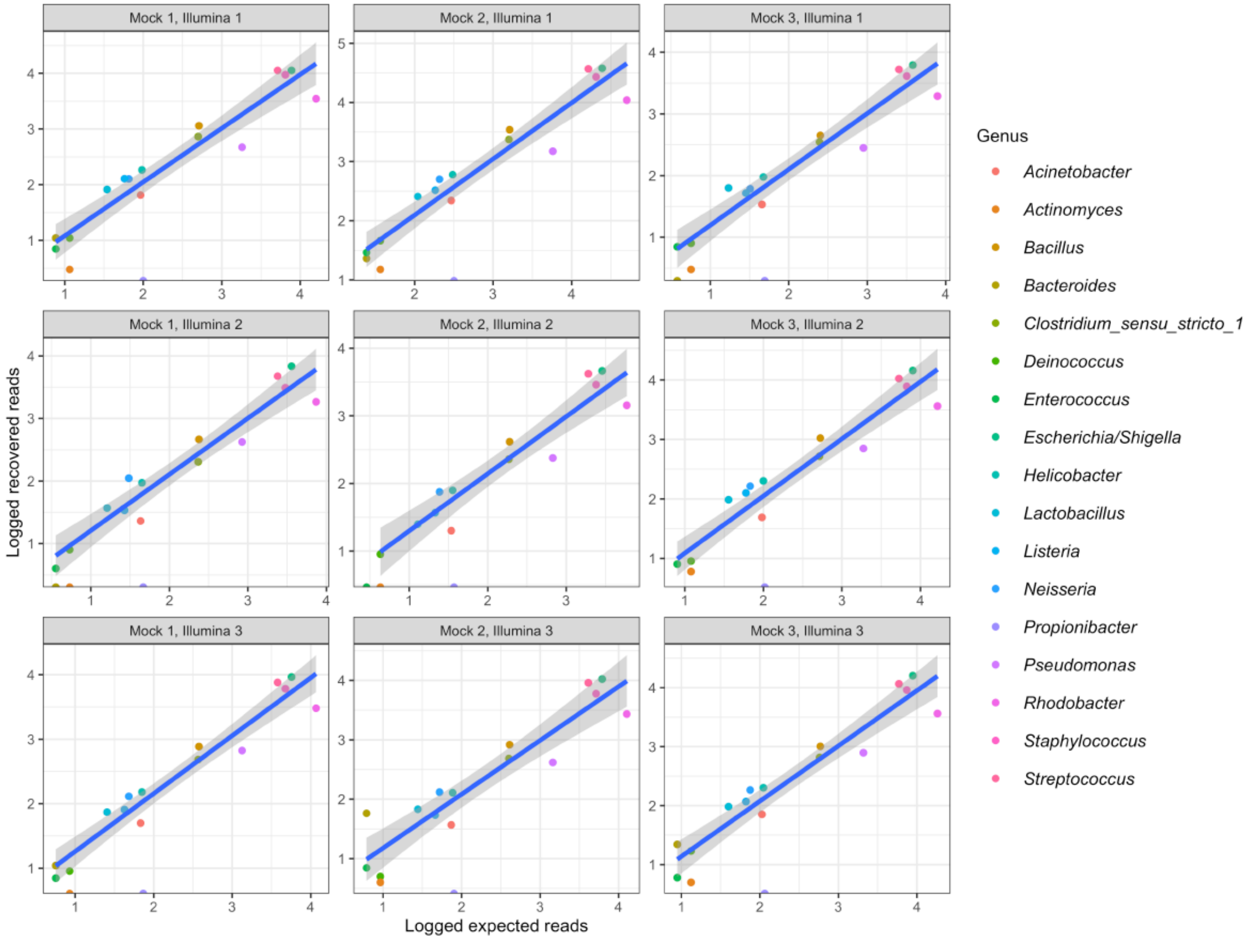

S1 Figure. Results of mock community analyses, showing the observed and expected mock community data. Three technical replicates of a tiered mock community were included in the three different Illumina runs that included samples analyzed in this manuscript.
