# Supplementary material for: Cloacal microbiomes of sympatric and allopatric Sceloporus lizards vary with environment and host relatedness
Source: PLoS One. 2022 Dec 22;17(12):e0279288. doi: 10.1371/journal.pone.0279288 (PMC9779040; doi:10.1371/journal.pone.0279288)
Supplement: S1 File — Analyses of alpha and beta diversity metrics for the cloacal microbiome of Sceloporus lizards. (PDF) [file pone.0279288.s002.pdf]

M. E. Bunker and S. L. Weiss

Cloacal microbiomes of sympatric and allopatric *Sceloporus* lizards vary with environment and host relatedness

Supporting Information: S1 File. Statistical output. Analyses of alpha and beta diversity metrics for the cloacal microbiome of *Sceloporus* lizards

PLOS ONE

Table A: Statistics comparing alpha diversity measures and post-hoc pairwise comparison between three *Sceloporus* species*: S. virgatus, S. jarrovii,* and *S. occidentalis*

**Comparison**	**DF**	**F value**	**P value**
Call: aov(log10(Shannon +1) ~ species			
species	2	31.26	< 0.001
Residuals	42		
Call: aov(log10(Observed) ~ species			
species	2	25.48	< 0.001
Residuals	42		
Call: aov(log10(PD) ~ species			
species	2	27.38	< 0.001
Residuals	42		

**Dunnet's Test (control = *S. virgatus)***	
**Comparison**	**P Value**
Shannon Diversity	
virgatus-jarrovii	0.983
virgatus-occidentalis	< 0.001
Richness	
virgatus-jarrovii	0.97
virgatus-occidentalis	< 0.001
PD	
virgatus-jarrovii	0.993
virgatus-occidentalis	<0.001

Table B: Statistics comparing community dispersion and composition based on three distance measures, with post-hoc pairwise comparison between three *Sceloporus* species*: S. virgatus, S. jarrovii,* and *S. occidentalis*

**Comparison**	**DF**	**F value**	**P value**
Betadispersion, Bray-Curtis distance			
species	2	21.95	< 0.001
Residuals	42		
Betadispersion, weighted UniFrac distance			
species	2	6.75	0.003
Residuals	42		
Betadispersion, unweighted UniFrac distance			
species	2	6.33	0.004
Residuals	42		

**Comparison**	**DF**	**F value**	**R^2**	**P value**
permANOVA, Bray-Curtis distance				
species	2	7.35	0.26	0.001
Residuals	42			
permANOVA, weighted UniFrac distance				
species	2	8.05	0.28	0.001
Residuals	75			
permANOVA, unweighted UniFrac distance				
species	2	7.92	0.27	0.001
Residuals	75			

**Pairwise Comparisons**				
**Comparison**	**DF**	**F**	**R^2**	**P.Adjusted**
Bray-Curtis				
virgatus-occidentalis	1	10.68	0.26	0.003
virgatus-jarrovii	1	4.18	0.13	0.003
weighted UniFrac				
virgatus-occidentalis	1	11.31	0.27	0.003
virgatus-jarrovii	1	2.51	0.08	0.135
unweighted UniFrac				
virgatus-occidentalis	1	13.25	0.31	0.003
virgatus-jarrovii	1	1.73	0.06	01026

Table C: Statistics comparing Alpha Diversity measures and post-hoc pairwise comparison between three *Sceloporus occidentalis* populations: Canyon, Forest, and Beach

Comparison	DF	F value	P value
Call: aov(Shannon ~ location * sex			
location	2	12.37	<0.001
sex	1	0.01	0.928
location:sex	2	2.17	0.121
Residuals	87		
Call: aov(log10(Observed) ~ location * sex			
location	2	12.39	<0.001
sex	1	0.03	0.874
location:sex	2	2.07	0.132
Residuals	87		
Call: aov(log10(PD) ~ location * sex			
location	2	10	< 0.001
sex	1	0.39	0.534
location:sex	2	1.75	0.180
Residuals	87		

Tukey Post-Hoc Test

Comparison	P Value
Shannon Diversity	
Canyon:Beach	< 0.001
Canyon:Forest	< 0.001
Beach:Forest	0.930
Richness	
Canyon:Beach	< 0.001
Canyon:Forest	< 0.001
Beach:Forest	0.878
PD	
Canyon:Beach	0.003
Canyon:Forest	<0.001
Beach:Forest	0.629

Table D: Statistics comparing community dispersion and composition based on three distance measures, with post-hoc pairwise comparison between three *Sceloporus occidentalis* populations: Canyon, Forest, and Beach

**Comparison**	**DF**	**F value**	**P value**
Betadispersion, Bray-Curtis distance			
location	2	5.53	0.005
sex	1	0.14	0.708
Residuals	90,91		
Betadispersion, weighted UniFrac distance			
location	2	2.68	0.074
sex	1	0.31	0.577
Residuals	90,91		
Betadispersion, unweighted UniFrac distance			
location	2	14.2	< 0.001
sex	1	1.02	0.315
Residuals	90,91		

**Comparison**	**DF**	**F value**	**R^2**	**P value**
permANOVA, Bray-Curtis distance				
location	2	5.73	0.11	0.001
sex	1	0.66	0.01	0.762
location * sex	2	1.85	0.04	0.022
Residuals	87			
permANOVA, weighted UniFrac distance				
location	2	3.29	0.07	0.007
sex	1	0.45	0	0.783
location * sex	2	1.30	0.03	0.223
Residuals	87			
permANOVA, unweighted UniFrac distance				
location	2	0.53	0.09	0.001
sex	1	4.42	0.01	0.87
location * sex	2	1.18	0.02	0.239

Pairwise Comparisons

**Comparison**	**DF**	**F**	**R^2**	**P.Adjusted**
Bray-Curtis				
Canyon:Beach	1	8.66	0.13	0.003
Canyon:Forest	1	7.66	0.12	0.003
Beach:Forest	1	1.76	0.03	0.186
weighted UniFrac				
Canyon:Beach	1	4.14	0.07	0.036
Canyon:Forest	1	5.29	0.09	0.012
Beach:Forest	1	1.32	0.02	0.678
unweighted UniFrac				
Canyon:Beach	1	5.78	0.09	0.003
Canyon:Forest	1	7.63	0.12	0.003
Beach:Forest	1	1.30	0.02	0.510
