# Supplementary material for: Cloacal microbiomes of sympatric and allopatric Sceloporus lizards vary with environment and host relatedness
Source: PLoS One. 2022 Dec 22;17(12):e0279288. doi: 10.1371/journal.pone.0279288 (PMC9779040; doi:10.1371/journal.pone.0279288)
Supplement: S2 File — (PDF) [file pone.0279288.s003.pdf]

M. E. Bunker and S. L. Weiss

Cloacal microbiomes of sympatric and allopatric *Sceloporus* lizards vary with environment and host relatedness

Supporting Information: S2 File. Relative abundances of the top 10 most abundant families and phyla in the cloacal microbiome of *Sceloporus* lizards

PLOS ONE

Relative abundance of top 10 most abundant families in three *Sceloporus* species

Sample	AnimalID	species	location	Enterobacteriaceae	Helicobacteraceae	Enterococcaceae	Corynebacteriaceae	Staphylococcaceae	Streptomycetaceae	Marinifilaceae	Bacteroidaceae	Tannerellaceae	Bacillaceae	Other
J06	1-8-11-20	jarrovi	AZ	0.248	0.010	0.131	0.017	0.000	0.000	0.095	0.100	0.055	0.000	0.345
J09	1-8-11-19	jarrovi	AZ	0.290	0.502	0.113	0.035	0.000	0.000	0.000	0.006	0.013	0.000	0.040
J15	NA8	jarrovi	AZ	0.283	0.276	0.172	0.103	0.000	0.000	0.001	0.005	0.002	0.000	0.158
J16	W7A	jarrovi	AZ	0.996	0.000	0.000	0.002	0.000	0.000	0.000	0.000	0.000	0.000	0.001
J17	W8A	jarrovi	AZ	0.196	0.683	0.038	0.064	0.000	0.000	0.000	0.000	0.000	0.000	0.018
J18	W9A	jarrovi	AZ	0.037	0.402	0.112	0.066	0.000	0.002	0.000	0.006	0.001	0.000	0.373
J21	W13A	jarrovi	AZ	0.078	0.851	0.025	0.038	0.000	0.000	0.001	0.000	0.001	0.000	0.006
J22	W15A	jarrovi	AZ	0.501	0.458	0.008	0.031	0.000	0.000	0.000	0.000	0.000	0.000	0.001
J23A	W19A	jarrovi	AZ	0.000	0.040	0.091	0.000	0.000	0.000	0.152	0.000	0.051	0.000	0.667
J26A	W24A	jarrovi	AZ	0.444	0.001	0.554	0.000	0.000	0.000	0.000	0.000	0.000	0.000	0.001
J27	W25A	jarrovi	AZ	0.962	0.000	0.033	0.005	0.000	0.000	0.000	0.000	0.000	0.000	0.000
J28A	W27A	jarrovi	AZ	0.584	0.115	0.147	0.094	0.000	0.000	0.004	0.019	0.007	0.000	0.030
J30A	W+A	jarrovi	AZ	0.421	0.158	0.000	0.380	0.000	0.006	0.000	0.017	0.000	0.000	0.019
			**Mean**	**0.388**	**0.269**	**0.110**	**0.064**	**0.000**	**0.001**	**0.019**	**0.012**	**0.010**	**0.000**	**0.128**
			**SD**	**0.316**	**0.287**	**0.146**	**0.101**	**0.000**	**0.002**	**0.048**	**0.027**	**0.019**	**0.000**	**0.208**
			**SE**	**0.088**	**0.080**	**0.040**	**0.028**	**0.000**	**0.000**	**0.013**	**0.008**	**0.005**	**0.000**	**0.058**
CB14	102	occi	Canyon	0.998	0.000	0.001	0.001	0.000	0.000	0.000	0.000	0.000	0.000	0.001
CB15	103	occi	Canyon	1.000	0.000	0.000	0.000	0.000	0.000	0.000	0.000	0.000	0.000	0.000
CB165	332	occi	Canyon	0.604	0.000	0.396	0.000	0.000	0.000	0.000	0.000	0.000	0.000	0.000
CB168	3002	occi	Canyon	0.935	0.000	0.000	0.000	0.000	0.000	0.000	0.000	0.000	0.065	0.000
CB169	421	occi	Canyon	0.754	0.000	0.243	0.000	0.000	0.000	0.000	0.000	0.000	0.003	0.000
CB170	313	occi	Canyon	0.644	0.000	0.356	0.000	0.000	0.000	0.000	0.000	0.000	0.000	0.000
CB172	423	occi	Canyon	1.000	0.000	0.000	0.000	0.000	0.000	0.000	0.000	0.000	0.000	0.000
CB174	3004	occi	Canyon	1.000	0.000	0.000	0.000	0.000	0.000	0.000	0.000	0.000	0.000	0.000
CB176	425	occi	Canyon	0.441	0.000	0.559	0.000	0.000	0.000	0.000	0.000	0.000	0.000	0.000
CB179	433	occi	Canyon	0.273	0.000	0.727	0.000	0.000	0.000	0.000	0.000	0.000	0.000	0.000
CB180	434	occi	Canyon	1.000	0.000	0.000	0.000	0.000	0.000	0.000	0.000	0.000	0.000	0.000
CB183	151	occi	Canyon	1.000	0.000	0.000	0.000	0.000	0.000	0.000	0.000	0.000	0.000	0.000
CB184	440	occi	Canyon	0.512	0.000	0.488	0.000	0.000	0.000	0.000	0.000	0.000	0.000	0.000
CB22	120	occi	Canyon	0.989	0.000	0.002	0.001	0.000	0.000	0.000	0.002	0.001	0.000	0.004
			**Mean**	**0.796**	**0.000**	**0.198**	**0.000**	**0.000**	**0.000**	**0.000**	**0.000**	**0.000**	**0.005**	**0.000**
			**SD**	**0.255**	**0.000**	**0.259**	**0.000**	**0.000**	**0.000**	**0.000**	**0.001**	**0.000**	**0.017**	**0.001**
			**SE**	**0.068**	**0.000**	**0.069**	**0.000**	**0.000**	**0.000**	**0.000**	**0.000**	**0.000**	**0.005**	**0.000**
F63	3145	virgatus	AZ	0.036	0.886	0.000	0.017	0.000	0.000	0.000	0.000	0.000	0.000	0.062
F64	3150	virgatus	AZ	0.297	0.008	0.000	0.000	0.000	0.000	0.281	0.042	0.208	0.000	0.164
F67	1101	virgatus	AZ	0.176	0.779	0.000	0.009	0.000	0.000	0.001	0.013	0.014	0.000	0.007
F76	3320	virgatus	AZ	0.313	0.684	0.000	0.000	0.000	0.000	0.000	0.000	0.002	0.000	0.001
F77	3321	virgatus	AZ	0.748	0.000	0.000	0.000	0.000	0.000	0.044	0.077	0.068	0.000	0.064
F78B	1340	virgatus	AZ	0.865	0.000	0.000	0.000	0.000	0.000	0.029	0.027	0.035	0.000	0.043
F79	3035	virgatus	AZ	0.000	0.994	0.000	0.003	0.000	0.000	0.001	0.000	0.001	0.000	0.001
F80B	3324	virgatus	AZ	0.981	0.001	0.000	0.000	0.000	0.000	0.002	0.004	0.000	0.000	0.011
V09	7037	virgatus	AZ	0.444	0.529	0.000	0.002	0.000	0.000	0.000	0.000	0.000	0.000	0.025
V10	NA5	virgatus	AZ	0.832	0.000	0.000	0.070	0.000	0.000	0.000	0.000	0.010	0.000	0.088
V11	W6C	virgatus	AZ	0.000	0.991	0.000	0.003	0.000	0.000	0.000	0.000	0.000	0.000	0.006
V13	W9C	virgatus	AZ	0.192	0.565	0.000	0.136	0.000	0.000	0.000	0.000	0.000	0.000	0.107
V14	5542	virgatus	AZ	0.998	0.000	0.000	0.000	0.000	0.000	0.000	0.000	0.001	0.000	0.001
V15	7115	virgatus	AZ	0.003	0.994	0.000	0.001	0.000	0.000	0.000	0.000	0.000	0.000	0.002
V16	7121	virgatus	AZ	0.010	0.875	0.010	0.081	0.000	0.000	0.000	0.000	0.000	0.000	0.025
V18	7122	virgatus	AZ	0.000	0.366	0.000	0.004	0.000	0.623	0.000	0.000	0.000	0.000	0.008
V19	7124	virgatus	AZ	0.981	0.000	0.000	0.002	0.000	0.002	0.000	0.000	0.000	0.000	0.015
V20	7130	virgatus	AZ	0.991	0.000	0.000	0.004	0.000	0.000	0.000	0.000	0.000	0.000	0.005
			**Mean**	**0.437**	**0.426**	**0.001**	**0.018**	**0.000**	**0.035**	**0.020**	**0.009**	**0.019**	**0.000**	**0.035**
			**SD**	**0.414**	**0.423**	**0.002**	**0.038**	**0.000**	**0.147**	**0.066**	**0.020**	**0.050**	**0.000**	**0.046**
			**SE**	**0.098**	**0.100**	**0.001**	**0.009**	**0.000**	**0.035**	**0.016**	**0.005**	**0.012**	**0.000**	**0.011**

Relative abundancesof top 10 most abundant families in three populations of *Sceloporus occidentalis* .

Sample	AnimalID	species	sex	location	Enterobacteriaceae	Helicobacteraceae	Enterococcaceae	Corynebacteriaceae	Staphylococcaceae	Streptomycetaceae	Marinifilaceae	Bacteroidaceae	Tannerellaceae	Bacillaceae	Other
CB14	102	occi	F	Canyon	0.998	0.000	0.001	0.001	0.000	0.000	0.000	0.000	0.000	0.000	0.001
CB15	103	occi	F	Canyon	1.000	0.000	0.000	0.000	0.000	0.000	0.000	0.000	0.000	0.000	0.000
CB165	332	occi	F	Canyon	0.604	0.000	0.396	0.000	0.000	0.000	0.000	0.000	0.000	0.000	0.000
CB168	3002	occi	F	Canyon	0.935	0.000	0.000	0.000	0.000	0.000	0.000	0.000	0.000	0.065	0.000
CB169	421	occi	F	Canyon	0.754	0.000	0.243	0.000	0.000	0.000	0.000	0.000	0.000	0.003	0.000
CB170	313	occi	F	Canyon	0.644	0.000	0.356	0.000	0.000	0.000	0.000	0.000	0.000	0.000	0.000
CB172	423	occi	F	Canyon	1.000	0.000	0.000	0.000	0.000	0.000	0.000	0.000	0.000	0.000	0.000
CB174	3004	occi	F	Canyon	1.000	0.000	0.000	0.000	0.000	0.000	0.000	0.000	0.000	0.000	0.000
CB176	425	occi	F	Canyon	0.441	0.000	0.559	0.000	0.000	0.000	0.000	0.000	0.000	0.000	0.000
CB179	433	occi	F	Canyon	0.273	0.000	0.727	0.000	0.000	0.000	0.000	0.000	0.000	0.000	0.000
CB180	434	occi	F	Canyon	1.000	0.000	0.000	0.000	0.000	0.000	0.000	0.000	0.000	0.000	0.000
CB183	151	occi	F	Canyon	1.000	0.000	0.000	0.000	0.000	0.000	0.000	0.000	0.000	0.000	0.000
CB184	440	occi	F	Canyon	0.512	0.000	0.488	0.000	0.000	0.000	0.000	0.000	0.000	0.000	0.000
CB22	120	occi	F	Canyon	0.989	0.000	0.002	0.001	0.000	0.000	0.000	0.002	0.001	0.000	0.004
CB16	105	occi	M	Canyon	1.000	0.000	0.000	0.000	0.000	0.000	0.000	0.000	0.000	0.000	0.000
CB167	122	occi	M	Canyon	0.000	0.000	0.000	0.000	0.000	0.000	0.000	0.000	0.000	1.000	0.000
CB171	3003a	occi	M	Canyon	0.304	0.000	0.696	0.000	0.000	0.000	0.000	0.000	0.000	0.000	0.000
CB175	424	occi	M	Canyon	1.000	0.000	0.000	0.000	0.000	0.000	0.000	0.000	0.000	0.000	0.000
CB182	453	occi	M	Canyon	0.464	0.000	0.536	0.000	0.000	0.000	0.000	0.000	0.000	0.000	0.000
CB186	443	occi	M	Canyon	0.691	0.000	0.309	0.000	0.000	0.000	0.000	0.000	0.000	0.000	0.000
CB187B	3402	occi	M	Canyon	0.189	0.000	0.794	0.000	0.000	0.000	0.002	0.007	0.003	0.000	0.006
CB188	420	occi	M	Canyon	0.577	0.000	0.422	0.000	0.000	0.000	0.000	0.000	0.000	0.001	0.000
CB30	133	occi	M	Canyon	0.998	0.000	0.000	0.000	0.000	0.000	0.000	0.000	0.000	0.000	0.001
				**Mean**	**0.712**	**0.000**	**0.240**	**0.000**	**0.000**	**0.000**	**0.000**	**0.000**	**0.000**	**0.047**	**0.001**
				**SD**	**0.318**	**0.000**	**0.283**	**0.000**	**0.000**	**0.000**	**0.000**	**0.002**	**0.001**	**0.208**	**0.001**
				**SE**	**0.066**	**0.000**	**0.059**	**0.000**	**0.000**	**0.000**	**0.000**	**0.000**	**0.000**	**0.043**	**0.000**
CB01	1051	occi	F	Beach	0.991	0.000	0.007	0.001	0.000	0.000	0.000	0.000	0.000	0.000	0.001
CB06	1101a	occi	F	Beach	0.966	0.000	0.004	0.005	0.001	0.000	0.006	0.004	0.005	0.000	0.009
CB07	1102a	occi	F	Beach	1.000	0.000	0.000	0.000	0.000	0.000	0.000	0.000	0.000	0.000	0.000
CB09	1104	occi	F	Beach	0.989	0.000	0.000	0.000	0.000	0.000	0.002	0.002	0.001	0.000	0.006
CB158	1414	occi	F	Beach	0.961	0.000	0.034	0.000	0.000	0.000	0.000	0.000	0.001	0.000	0.004
CB159	2021	occi	F	Beach	0.992	0.000	0.000	0.000	0.000	0.000	0.000	0.001	0.001	0.000	0.006
CB200	2143	occi	F	Beach	0.894	0.000	0.062	0.000	0.000	0.000	0.000	0.000	0.000	0.000	0.044
CB201	2144	occi	F	Beach	0.972	0.000	0.028	0.000	0.000	0.000	0.000	0.000	0.000	0.000	0.000
CB202	2145	occi	F	Beach	0.993	0.000	0.003	0.004	0.000	0.000	0.000	0.000	0.000	0.000	0.000
CB203	2150	occi	F	Beach	0.999	0.000	0.001	0.000	0.000	0.000	0.000	0.000	0.000	0.000	0.000
CB208	2153a	occi	F	Beach	0.998	0.000	0.000	0.000	0.000	0.000	0.000	0.000	0.001	0.000	0.001
CB209	2155	occi	F	Beach	0.986	0.000	0.004	0.000	0.009	0.000	0.000	0.000	0.000	0.000	0.000
CB210	2301	occi	F	Beach	1.000	0.000	0.000	0.000	0.000	0.000	0.000	0.000	0.000	0.000	0.000
CB211	2302	occi	F	Beach	0.996	0.000	0.004	0.000	0.000	0.000	0.000	0.000	0.000	0.000	0.000
CB212	2303	occi	F	Beach	0.999	0.000	0.000	0.000	0.000	0.000	0.000	0.000	0.000	0.000	0.001
CB213	2304	occi	F	Beach	0.954	0.000	0.001	0.000	0.000	0.000	0.020	0.009	0.003	0.000	0.013
CB215	2311	occi	F	Beach	0.981	0.000	0.005	0.000	0.000	0.000	0.000	0.000	0.000	0.000	0.014
CB34	NA2	occi	F	Beach	1.000	0.000	0.000	0.000	0.000	0.000	0.000	0.000	0.000	0.000	0.000
CB35	NA3	occi	F	Beach	0.998	0.000	0.000	0.000	0.000	0.000	0.000	0.000	0.000	0.000	0.001
CB36	NA4	occi	F	Beach	0.967	0.000	0.005	0.023	0.000	0.000	0.000	0.001	0.000	0.000	0.002
CB39	1112b	occi	F	Beach	0.976	0.000	0.000	0.018	0.000	0.000	0.001	0.000	0.002	0.000	0.003
CB02	1052	occi	M	Beach	0.995	0.000	0.000	0.002	0.000	0.000	0.001	0.000	0.000	0.000	0.002
CB10	15230	occi	M	Beach	0.946	0.000	0.004	0.010	0.000	0.000	0.001	0.000	0.004	0.000	0.035
CB161	1044	occi	M	Beach	0.923	0.000	0.003	0.053	0.000	0.000	0.000	0.000	0.000	0.000	0.020
CB162	2012	occi	M	Beach	0.971	0.000	0.001	0.000	0.000	0.000	0.000	0.001	0.000	0.000	0.027
CB205	2152	occi	M	Beach	1.000	0.000	0.000	0.000	0.000	0.000	0.000	0.000	0.000	0.000	0.000
CB206	1041	occi	M	Beach	0.866	0.000	0.000	0.006	0.110	0.000	0.000	0.000	0.000	0.000	0.018
CB207	2009	occi	M	Beach	0.996	0.000	0.003	0.000	0.000	0.000	0.000	0.000	0.000	0.000	0.000
CB214	2305	occi	M	Beach	0.993	0.000	0.000	0.000	0.000	0.000	0.002	0.002	0.001	0.000	0.001
CB216	2312	occi	M	Beach	0.996	0.000	0.001	0.000	0.000	0.000	0.001	0.000	0.000	0.000	0.002
CB218	2314	occi	M	Beach	0.999	0.000	0.000	0.000	0.000	0.000	0.001	0.000	0.000	0.000	0.000
CB227	2330	occi	M	Beach	0.977	0.000	0.023	0.000	0.000	0.000	0.000	0.000	0.000	0.000	0.000
CB279	2331	occi	M	Beach	0.942	0.000	0.002	0.000	0.010	0.000	0.011	0.005	0.009	0.000	0.021
CB33	NA1	occi	M	Beach	0.997	0.000	0.000	0.000	0.000	0.000	0.000	0.000	0.000	0.000	0.001
CB38	1111b	occi	M	Beach	0.995	0.000	0.000	0.000	0.000	0.000	0.001	0.000	0.000	0.000	0.004

CB40	1113	occi	M	Beach	0.987	0.000	0.000	0.001	0.005	0.000	0.001	0.000	0.002	0.000	0.005
CB41	1114	occi	M	Beach	0.989	0.000	0.001	0.002	0.001	0.000	0.000	0.000	0.000	0.004	0.003
				**Mean**	**0.978**	**0.000**	**0.005**	**0.003**	**0.004**	**0.000**	**0.001**	**0.001**	**0.001**	**0.000**	**0.007**
				**SD**	**0.030**	**0.000**	**0.012**	**0.010**	**0.018**	**0.000**	**0.004**	**0.002**	**0.002**	**0.001**	**0.011**
				**SE**	**0.005**	**0.000**	**0.002**	**0.002**	**0.003**	**0.000**	**0.001**	**0.000**	**0.000**	**0.000**	**0.002**
CB190	2131	occi	F	Forest	0.668	0.000	0.149	0.001	0.178	0.000	0.000	0.000	0.000	0.000	0.002
CB198	2141	occi	F	Forest	0.722	0.000	0.098	0.138	0.010	0.000	0.000	0.000	0.000	0.000	0.033
CB221	2090	occi	F	Forest	0.982	0.000	0.007	0.003	0.007	0.000	0.000	0.000	0.000	0.000	0.001
CB222	3090	occi	F	Forest	0.980	0.000	0.005	0.011	0.001	0.000	0.001	0.000	0.000	0.000	0.002
CB223	2322	occi	F	Forest	0.999	0.000	0.000	0.000	0.000	0.000	0.001	0.000	0.000	0.000	0.000
CB225	2324	occi	F	Forest	0.999	0.000	0.000	0.000	0.000	0.000	0.000	0.000	0.001	0.000	0.000
CB287	2404	occi	F	Forest	0.998	0.000	0.000	0.002	0.000	0.000	0.000	0.000	0.000	0.000	0.000
CB289	2405	occi	F	Forest	0.973	0.000	0.001	0.000	0.000	0.000	0.004	0.007	0.002	0.000	0.012
CB44	1121	occi	F	Forest	0.990	0.000	0.002	0.000	0.000	0.000	0.000	0.002	0.001	0.000	0.005
CB54	1401	occi	F	Forest	0.981	0.000	0.011	0.008	0.000	0.000	0.000	0.000	0.000	0.000	0.000
CB55	1133	occi	F	Forest	0.916	0.000	0.058	0.026	0.000	0.000	0.000	0.000	0.000	0.000	0.001
CB56	1132	occi	F	Forest	0.921	0.000	0.006	0.004	0.000	0.000	0.026	0.007	0.013	0.000	0.022
CB57	1134	occi	F	Forest	0.970	0.000	0.002	0.006	0.000	0.000	0.006	0.007	0.001	0.000	0.009
CB59	1135	occi	F	Forest	0.993	0.000	0.001	0.000	0.000	0.000	0.002	0.000	0.001	0.000	0.004
CB61	1141	occi	F	Forest	0.985	0.000	0.000	0.000	0.000	0.000	0.005	0.004	0.000	0.000	0.006
CB189	2130	occi	M	Forest	1.000	0.000	0.000	0.000	0.000	0.000	0.000	0.000	0.000	0.000	0.000
CB191	2132	occi	M	Forest	0.962	0.000	0.001	0.000	0.002	0.000	0.005	0.010	0.006	0.000	0.013
CB194	1341	occi	M	Forest	0.608	0.000	0.352	0.040	0.000	0.000	0.000	0.000	0.000	0.000	0.000
CB196	2140	occi	M	Forest	0.985	0.000	0.014	0.000	0.000	0.000	0.000	0.000	0.000	0.000	0.000
CB197	1123	occi	M	Forest	0.990	0.000	0.010	0.000	0.000	0.000	0.000	0.000	0.000	0.000	0.000
CB199	2142	occi	M	Forest	0.925	0.000	0.061	0.005	0.009	0.000	0.000	0.000	0.000	0.000	0.000
CB220	2320	occi	M	Forest	1.000	0.000	0.000	0.000	0.000	0.000	0.000	0.000	0.000	0.000	0.000
CB224	2323	occi	M	Forest	0.999	0.000	0.000	0.000	0.000	0.000	0.000	0.000	0.000	0.000	0.000
CB281	2333	occi	M	Forest	0.998	0.000	0.002	0.000	0.000	0.000	0.000	0.000	0.000	0.000	0.000
CB282	1312	occi	M	Forest	0.953	0.000	0.007	0.000	0.000	0.000	0.002	0.014	0.008	0.000	0.016
CB42	1115a	occi	M	Forest	0.996	0.000	0.000	0.000	0.000	0.000	0.000	0.000	0.000	0.000	0.004
CB43	1120	occi	M	Forest	0.994	0.000	0.002	0.001	0.000	0.000	0.000	0.000	0.000	0.000	0.002
CB45	1122	occi	M	Forest	0.997	0.000	0.003	0.000	0.000	0.000	0.000	0.000	0.000	0.000	0.001
CB46	1130	occi	M	Forest	0.997	0.000	0.001	0.001	0.000	0.000	0.000	0.000	0.000	0.000	0.000
CB49	1151	occi	M	Forest	0.991	0.000	0.002	0.002	0.000	0.000	0.001	0.002	0.000	0.000	0.003
CB50	5012	occi	M	Forest	0.849	0.000	0.126	0.001	0.000	0.000	0.004	0.002	0.004	0.000	0.014
CB51	1124	occi	M	Forest	0.991	0.000	0.006	0.002	0.000	0.000	0.000	0.000	0.001	0.000	0.001
CB53	1131	occi	M	Forest	0.999	0.000	0.000	0.000	0.000	0.000	0.000	0.000	0.000	0.000	0.000
				**Mean**	**0.949**	**0.000**	**0.028**	**0.008**	**0.006**	**0.000**	**0.002**	**0.002**	**0.001**	**0.000**	**0.005**
				**SD**	**0.097**	**0.000**	**0.069**	**0.025**	**0.031**	**0.000**	**0.005**	**0.003**	**0.003**	**0.000**	**0.008**
				**SE**	**0.017**	**0.000**	**0.012**	**0.004**	**0.005**	**0.000**	**0.001**	**0.001**	**0.000**	**0.000**	**0.001**

Relative abundance of top 10 most abundant phyla in three *Sceloporus* species

Sample	Animal ID	species	Proteo + Epsilon	Proteobacteria	Epsilonbacteraeota	Firmicutes	Actinobacteria	Bacteroidetes	Parabasalia	Verrucomicrobia	Cyanobacteria	Chlamydiae	Unknown	Other
J06	1-8-11-20	jarrovi	0.312	0.302	0.010	0.338	0.079	0.267	0.000	0.000	0.005	0.000	0.000	0.000
J09	1-8-11-19	jarrovi	0.808	0.306	0.502	0.136	0.035	0.021	0.000	0.000	0.000	0.001	0.000	0.000
J15	NA8	jarrovi	0.673	0.397	0.276	0.211	0.105	0.008	0.000	0.000	0.001	0.000	0.000	0.001
J16	W7A	jarrovi	0.996	0.996	0.000	0.002	0.002	0.000	0.000	0.000	0.000	0.000	0.000	0.000
J17	W8A	jarrovi	0.882	0.200	0.683	0.041	0.075	0.001	0.000	0.000	0.001	0.000	0.000	0.000
J18	W9A	jarrovi	0.441	0.039	0.402	0.125	0.072	0.008	0.355	0.000	0.000	0.000	0.000	0.001
J21	W13A	jarrovi	0.930	0.078	0.851	0.030	0.038	0.002	0.000	0.000	0.000	0.000	0.000	0.000
J22	W15A	jarrovi	0.960	0.502	0.458	0.008	0.031	0.000	0.000	0.000	0.000	0.000	0.000	0.000
J23A	W19A	jarrovi	0.253	0.212	0.040	0.374	0.000	0.374	0.000	0.000	0.000	0.000	0.000	0.000
J26A	W24A	jarrovi	0.445	0.444	0.001	0.554	0.000	0.001	0.000	0.000	0.000	0.000	0.000	0.000
J27	W25A	jarrovi	0.962	0.962	0.000	0.033	0.005	0.000	0.000	0.000	0.000	0.000	0.000	0.000
J28A	W27A	jarrovi	0.703	0.588	0.115	0.173	0.094	0.030	0.000	0.000	0.000	0.000	0.000	0.000
J30A	W+A	jarrovi	0.584	0.427	0.158	0.000	0.399	0.017	0.000	0.000	0.000	0.000	0.000	0.000
		**Mean**	**0.688**	**0.419**	**0.269**	**0.156**	**0.072**	**0.056**	**0.027**	**0.000**	**0.001**	**0.000**	**0.000**	**0.000**
		**SD**	**0.261**	**0.295**	**0.287**	**0.173**	**0.105**	**0.120**	**0.098**	**0.000**	**0.001**	**0.000**	**0.000**	**0.000**
		**SE**	**0.072**	**0.082**	**0.080**	**0.048**	**0.029**	**0.033**	**0.027**	**0.000**	**0.000**	**0.000**	**0.000**	**0.000**
CB14	102	occi	0.998	0.998	0.000	0.001	0.001	0.000	0.001	0.000	0.000	0.000	0.000	0.000
CB15	103	occi	1.000	1.000	0.000	0.000	0.000	0.000	0.000	0.000	0.000	0.000	0.000	0.000
CB165	332	occi	0.604	0.604	0.000	0.396	0.000	0.000	0.000	0.000	0.000	0.000	0.000	0.000
CB168	3002	occi	0.935	0.935	0.000	0.065	0.000	0.000	0.000	0.000	0.000	0.000	0.000	0.000
CB169	421	occi	0.754	0.754	0.000	0.246	0.000	0.000	0.000	0.000	0.000	0.000	0.000	0.000
CB170	313	occi	0.644	0.644	0.000	0.356	0.000	0.000	0.000	0.000	0.000	0.000	0.000	0.000
CB172	423	occi	1.000	1.000	0.000	0.000	0.000	0.000	0.000	0.000	0.000	0.000	0.000	0.000
CB174	3004	occi	1.000	1.000	0.000	0.000	0.000	0.000	0.000	0.000	0.000	0.000	0.000	0.000
CB176	425	occi	0.441	0.441	0.000	0.559	0.000	0.000	0.000	0.000	0.000	0.000	0.000	0.000
CB179	433	occi	0.273	0.273	0.000	0.727	0.000	0.000	0.000	0.000	0.000	0.000	0.000	0.000
CB180	434	occi	1.000	1.000	0.000	0.000	0.000	0.000	0.000	0.000	0.000	0.000	0.000	0.000
CB183	151	occi	1.000	1.000	0.000	0.000	0.000	0.000	0.000	0.000	0.000	0.000	0.000	0.000
CB184	440	occi	0.512	0.512	0.000	0.488	0.000	0.000	0.000	0.000	0.000	0.000	0.000	0.000
CB22	120	occi	0.990	0.990	0.000	0.005	0.001	0.004	0.000	0.000	0.000	0.000	0.000	0.000
		**Mean**	**0.797**	**0.797**	**0.000**	**0.203**	**0.000**	**0.000**	**0.000**	**0.000**	**0.000**	**0.000**	**0.000**	**0.000**
		**SD**	**0.255**	**0.255**	**0.000**	**0.256**	**0.000**	**0.001**	**0.000**	**0.000**	**0.000**	**0.000**	**0.000**	**0.000**
		**SE**	**0.068**	**0.068**	**0.000**	**0.068**	**0.000**	**0.000**	**0.000**	**0.000**	**0.000**	**0.000**	**0.000**	**0.000**
F63	3145	virgatus	0.960	0.074	0.886	0.001	0.033	0.006	0.000	0.000	0.000	0.000	0.000	0.000
F64	3150	virgatus	0.333	0.326	0.008	0.058	0.003	0.601	0.000	0.004	0.000	0.000	0.000	0.000
F67	1101	virgatus	0.960	0.180	0.779	0.003	0.009	0.028	0.000	0.000	0.000	0.000	0.000	0.000
F76	3320	virgatus	0.997	0.313	0.684	0.001	0.000	0.002	0.000	0.000	0.000	0.000	0.000	0.000
F77	3321	virgatus	0.749	0.749	0.000	0.038	0.000	0.212	0.000	0.000	0.001	0.000	0.000	0.000
F78B	1340	virgatus	0.870	0.870	0.000	0.027	0.000	0.100	0.000	0.002	0.000	0.000	0.000	0.000
F79	3035	virgatus	0.994	0.000	0.994	0.001	0.003	0.002	0.000	0.000	0.000	0.000	0.000	0.000
F80B	3324	virgatus	0.986	0.985	0.001	0.002	0.003	0.008	0.000	0.000	0.000	0.000	0.000	0.002
V09	7037	virgatus	0.987	0.458	0.529	0.001	0.012	0.000	0.000	0.000	0.000	0.000	0.000	0.000
V10	NA5	virgatus	0.873	0.873	0.000	0.007	0.096	0.021	0.000	0.000	0.003	0.000	0.000	0.000
V11	W6C	virgatus	0.994	0.003	0.991	0.000	0.003	0.000	0.000	0.000	0.000	0.002	0.000	0.000
V13	W9C	virgatus	0.821	0.256	0.565	0.025	0.143	0.010	0.000	0.000	0.000	0.000	0.000	0.000
V14	5542	virgatus	0.998	0.998	0.000	0.000	0.000	0.001	0.000	0.000	0.000	0.000	0.000	0.000
V15	7115	virgatus	0.999	0.004	0.994	0.000	0.001	0.000	0.000	0.000	0.000	0.000	0.000	0.000
V16	7121	virgatus	0.898	0.023	0.875	0.010	0.084	0.000	0.000	0.000	0.002	0.006	0.000	0.000
V18	7122	virgatus	0.369	0.003	0.366	0.004	0.627	0.001	0.000	0.000	0.000	0.000	0.000	0.000
V19	7124	virgatus	0.989	0.989	0.000	0.002	0.007	0.001	0.000	0.000	0.000	0.000	0.000	0.000
V20	7130	virgatus	0.996	0.996	0.000	0.000	0.004	0.001	0.000	0.000	0.000	0.000	0.000	0.000
		**Mean**	**0.876**	**0.450**	**0.426**	**0.010**	**0.057**	**0.055**	**0.000**	**0.000**	**0.000**	**0.000**	**0.000**	**0.000**
		**SD**	**0.204**	**0.412**	**0.423**	**0.016**	**0.148**	**0.146**	**0.000**	**0.001**	**0.001**	**0.001**	**0.000**	**0.000**
		**SE**	**0.048**	**0.097**	**0.100**	**0.004**	**0.035**	**0.034**	**0.000**	**0.000**	**0.000**	**0.000**	**0.000**	**0.000**

Relative abundance of top 10 most abundant phyla in three populations of *Sceloporus occidentalis*

Sample	toe.clip	sex	location	Prote + Epsilon	Proteobacteria	Epsilonbacteraeota	Firmicutes	Actinobacteria	Bacteroidetes	Parabasalia	Verrucomicrobia	Cyanobacteria	Chlamydiae	Unknown	Other
CB14	102	F	Canyon	0.998	0.998	0.000	0.001	0.001	0.000	0.001	0.000	0.000	0.000	0.000	0.000
CB15	103	F	Canyon	1.000	1.000	0.000	0.000	0.000	0.000	0.000	0.000	0.000	0.000	0.000	0.000
CB165	332	F	Canyon	0.604	0.604	0.000	0.396	0.000	0.000	0.000	0.000	0.000	0.000	0.000	0.000
CB168	3002	F	Canyon	0.935	0.935	0.000	0.065	0.000	0.000	0.000	0.000	0.000	0.000	0.000	0.000
CB169	421	F	Canyon	0.754	0.754	0.000	0.246	0.000	0.000	0.000	0.000	0.000	0.000	0.000	0.000
CB170	313	F	Canyon	0.644	0.644	0.000	0.356	0.000	0.000	0.000	0.000	0.000	0.000	0.000	0.000
CB172	423	F	Canyon	1.000	1.000	0.000	0.000	0.000	0.000	0.000	0.000	0.000	0.000	0.000	0.000
CB174	3004	F	Canyon	1.000	1.000	0.000	0.000	0.000	0.000	0.000	0.000	0.000	0.000	0.000	0.000
CB176	425	F	Canyon	0.441	0.441	0.000	0.559	0.000	0.000	0.000	0.000	0.000	0.000	0.000	0.000
CB179	433	F	Canyon	0.273	0.273	0.000	0.727	0.000	0.000	0.000	0.000	0.000	0.000	0.000	0.000
CB180	434	F	Canyon	1.000	1.000	0.000	0.000	0.000	0.000	0.000	0.000	0.000	0.000	0.000	0.000
CB183	151	F	Canyon	1.000	1.000	0.000	0.000	0.000	0.000	0.000	0.000	0.000	0.000	0.000	0.000
CB184	440	F	Canyon	0.512	0.512	0.000	0.488	0.000	0.000	0.000	0.000	0.000	0.000	0.000	0.000
CB22	120	F	Canyon	0.990	0.990	0.000	0.005	0.001	0.004	0.000	0.000	0.000	0.000	0.000	0.000
CB16	105	M	Canyon	1.000	1.000	0.000	0.000	0.000	0.000	0.000	0.000	0.000	0.000	0.000	0.000
CB167	122	M	Canyon	0.000	0.000	0.000	1.000	0.000	0.000	0.000	0.000	0.000	0.000	0.000	0.000
CB171	3003a	M	Canyon	0.304	0.304	0.000	0.696	0.000	0.000	0.000	0.000	0.000	0.000	0.000	0.000
CB175	424	M	Canyon	1.000	1.000	0.000	0.000	0.000	0.000	0.000	0.000	0.000	0.000	0.000	0.000
CB182	453	M	Canyon	0.464	0.464	0.000	0.536	0.000	0.000	0.000	0.000	0.000	0.000	0.000	0.000
CB186	443	M	Canyon	0.691	0.691	0.000	0.309	0.000	0.000	0.000	0.000	0.000	0.000	0.000	0.000
CB187B	3402	M	Canyon	0.190	0.190	0.000	0.795	0.000	0.013	0.000	0.001	0.000	0.000	0.000	0.000
CB188	420	M	Canyon	0.577	0.577	0.000	0.423	0.000	0.000	0.000	0.000	0.000	0.000	0.000	0.000
CB30	133	M	Canyon	0.998	0.998	0.000	0.001	0.000	0.000	0.000	0.000	0.000	0.000	0.000	0.000
			**Mean**	**0.712**	**0.712**	**0.000**	**0.287**	**0.000**	**0.001**	**0.000**	**0.000**	**0.000**	**0.000**	**0.000**	**0.000**
			**SD**	**0.318**	**0.318**	**0.000**	**0.317**	**0.000**	**0.003**	**0.000**	**0.000**	**0.000**	**0.000**	**0.000**	**0.000**
			**SE**	**0.066**	**0.066**	**0.000**	**0.066**	**0.000**	**0.001**	**0.000**	**0.000**	**0.000**	**0.000**	**0.000**	**0.000**
CB01	1051	F	Beach	0.991	0.991	0.000	0.007	0.001	0.001	0.000	0.000	0.000	0.000	0.000	0.000
CB06	1101a	F	Beach	0.966	0.966	0.000	0.010	0.005	0.017	0.000	0.002	0.000	0.000	0.000	0.000
CB07	1102a	F	Beach	1.000	1.000	0.000	0.000	0.000	0.000	0.000	0.000	0.000	0.000	0.000	0.000
CB09	1104	F	Beach	0.989	0.989	0.000	0.005	0.000	0.006	0.000	0.000	0.000	0.000	0.000	0.000
CB158	1414	F	Beach	0.962	0.961	0.000	0.037	0.001	0.001	0.000	0.000	0.000	0.000	0.000	0.000
CB159	2021	F	Beach	0.993	0.992	0.000	0.004	0.000	0.003	0.000	0.000	0.000	0.000	0.000	0.000
CB200	2143	F	Beach	0.894	0.894	0.000	0.106	0.000	0.000	0.000	0.000	0.000	0.000	0.000	0.000
CB201	2144	F	Beach	0.972	0.972	0.000	0.028	0.000	0.000	0.000	0.000	0.000	0.000	0.000	0.000
CB202	2145	F	Beach	0.993	0.993	0.000	0.003	0.004	0.000	0.000	0.000	0.000	0.000	0.000	0.000
CB203	2150	F	Beach	0.999	0.999	0.000	0.001	0.000	0.000	0.000	0.000	0.000	0.000	0.000	0.000
CB208	2153a	F	Beach	0.998	0.998	0.000	0.001	0.000	0.002	0.000	0.000	0.000	0.000	0.000	0.000
CB209	2155	F	Beach	0.986	0.986	0.000	0.014	0.000	0.000	0.000	0.000	0.000	0.000	0.000	0.000
CB210	2301	F	Beach	1.000	1.000	0.000	0.000	0.000	0.000	0.000	0.000	0.000	0.000	0.000	0.000
CB211	2302	F	Beach	0.996	0.996	0.000	0.004	0.000	0.000	0.000	0.000	0.000	0.000	0.000	0.000
CB212	2303	F	Beach	1.000	1.000	0.000	0.000	0.000	0.000	0.000	0.000	0.000	0.000	0.000	0.000
CB213	2304	F	Beach	0.954	0.954	0.000	0.010	0.000	0.035	0.000	0.001	0.000	0.000	0.000	0.000
CB215	2311	F	Beach	0.995	0.995	0.000	0.005	0.000	0.000	0.000	0.000	0.000	0.000	0.000	0.000
CB34	NA2	F	Beach	1.000	1.000	0.000	0.000	0.000	0.000	0.000	0.000	0.000	0.000	0.000	0.000
CB35	NA3	F	Beach	0.998	0.998	0.000	0.001	0.000	0.000	0.000	0.000	0.000	0.000	0.000	0.000
CB36	NA4	F	Beach	0.967	0.967	0.000	0.007	0.023	0.002	0.000	0.000	0.000	0.000	0.000	0.000
CB39	1112b	F	Beach	0.976	0.976	0.000	0.003	0.018	0.002	0.000	0.000	0.001	0.000	0.000	0.000
CB02	1052	M	Beach	0.995	0.995	0.000	0.001	0.002	0.001	0.000	0.000	0.000	0.000	0.000	0.000
CB10	15230	M	Beach	0.952	0.952	0.000	0.013	0.010	0.008	0.014	0.000	0.003	0.000	0.000	0.000
CB161	1044	M	Beach	0.938	0.938	0.000	0.003	0.058	0.001	0.000	0.000	0.000	0.000	0.000	0.000
CB162	2012	M	Beach	0.980	0.977	0.004	0.002	0.000	0.004	0.000	0.001	0.000	0.000	0.003	0.000

CB205	2152	M	Beach	1.000	1.000	0.000	0.000	0.000	0.000	0.000	0.000	0.000	0.000	0.000	0.000
CB206	1041	M	Beach	0.884	0.884	0.000	0.110	0.006	0.000	0.000	0.000	0.000	0.000	0.000	0.000
CB207	2009	M	Beach	0.996	0.996	0.000	0.003	0.000	0.000	0.000	0.000	0.000	0.000	0.000	0.000
CB214	2305	M	Beach	0.993	0.993	0.000	0.001	0.000	0.006	0.000	0.000	0.000	0.000	0.000	0.000
CB216	2312	M	Beach	0.996	0.996	0.000	0.002	0.000	0.002	0.000	0.000	0.000	0.000	0.000	0.000
CB218	2314	M	Beach	0.999	0.999	0.000	0.000	0.000	0.001	0.000	0.000	0.000	0.000	0.000	0.000
CB227	2330	M	Beach	0.977	0.977	0.000	0.023	0.000	0.000	0.000	0.000	0.000	0.000	0.000	0.000
CB279	2331	M	Beach	0.947	0.947	0.000	0.022	0.000	0.028	0.000	0.003	0.000	0.000	0.000	0.000
CB33	NA1	M	Beach	0.998	0.998	0.000	0.001	0.000	0.001	0.000	0.000	0.000	0.000	0.000	0.000
CB38	1111b	M	Beach	0.996	0.996	0.000	0.003	0.000	0.002	0.000	0.000	0.000	0.000	0.000	0.000
CB40	1113	M	Beach	0.987	0.987	0.000	0.010	0.001	0.003	0.000	0.000	0.000	0.000	0.000	0.000
CB41	1114	M	Beach	0.991	0.991	0.000	0.007	0.002	0.000	0.000	0.000	0.000	0.000	0.000	0.000
			**Mean**	**0.980**	**0.980**	**0.000**	**0.012**	**0.004**	**0.003**	**0.000**	**0.000**	**0.000**	**0.000**	**0.000**	**0.000**
			**SD**	**0.028**	**0.028**	**0.001**	**0.025**	**0.010**	**0.008**	**0.002**	**0.001**	**0.001**	**0.000**	**0.001**	**0.000**
			**SE**	**0.005**	**0.005**	**0.000**	**0.004**	**0.002**	**0.001**	**0.000**	**0.000**	**0.000**	**0.000**	**0.000**	**0.000**
CB190	2131	F	Forest	0.668	0.668	0.000	0.328	0.001	0.001	0.000	0.000	0.000	0.000	0.000	0.000
CB198	2141	F	Forest	0.742	0.742	0.000	0.108	0.149	0.001	0.000	0.000	0.000	0.000	0.000	0.000
CB221	2090	F	Forest	0.982	0.982	0.000	0.014	0.004	0.000	0.000	0.000	0.000	0.000	0.000	0.000
CB222	3090	F	Forest	0.980	0.980	0.000	0.006	0.013	0.001	0.000	0.000	0.000	0.000	0.000	0.000
CB223	2322	F	Forest	0.999	0.999	0.000	0.000	0.000	0.001	0.000	0.000	0.000	0.000	0.000	0.000
CB225	2324	F	Forest	0.999	0.999	0.000	0.000	0.000	0.001	0.000	0.000	0.000	0.000	0.000	0.000
CB287	2404	F	Forest	0.998	0.998	0.000	0.000	0.002	0.000	0.000	0.000	0.000	0.000	0.000	0.000
CB289	2405	F	Forest	0.974	0.974	0.000	0.010	0.000	0.015	0.002	0.000	0.000	0.000	0.000	0.000
CB44	1121	F	Forest	0.991	0.991	0.000	0.005	0.000	0.003	0.000	0.000	0.000	0.000	0.000	0.000
CB54	1401	F	Forest	0.981	0.981	0.000	0.011	0.008	0.000	0.000	0.000	0.000	0.000	0.000	0.000
CB55	1133	F	Forest	0.916	0.916	0.000	0.058	0.026	0.000	0.000	0.000	0.001	0.000	0.000	0.000
CB56	1132	F	Forest	0.923	0.923	0.000	0.018	0.004	0.053	0.000	0.001	0.000	0.000	0.000	0.000
CB57	1134	F	Forest	0.970	0.970	0.000	0.008	0.006	0.015	0.000	0.000	0.001	0.000	0.000	0.000
CB59	1135	F	Forest	0.995	0.995	0.000	0.002	0.000	0.003	0.000	0.000	0.000	0.000	0.000	0.000
CB61	1141	F	Forest	0.985	0.985	0.000	0.003	0.001	0.009	0.000	0.000	0.001	0.000	0.000	0.000
CB189	2130	M	Forest	1.000	1.000	0.000	0.000	0.000	0.000	0.000	0.000	0.000	0.000	0.000	0.000
CB191	2132	M	Forest	0.962	0.962	0.000	0.012	0.000	0.025	0.000	0.001	0.000	0.000	0.000	0.000
CB194	1341	M	Forest	0.608	0.608	0.000	0.352	0.040	0.000	0.000	0.000	0.000	0.000	0.000	0.000
CB196	2140	M	Forest	0.985	0.985	0.000	0.014	0.000	0.000	0.000	0.000	0.000	0.000	0.000	0.000
CB197	1123	M	Forest	0.990	0.990	0.000	0.010	0.000	0.000	0.000	0.000	0.000	0.000	0.000	0.000
CB199	2142	M	Forest	0.925	0.925	0.000	0.070	0.005	0.000	0.000	0.000	0.000	0.000	0.000	0.000
CB220	2320	M	Forest	1.000	1.000	0.000	0.000	0.000	0.000	0.000	0.000	0.000	0.000	0.000	0.000
CB224	2323	M	Forest	0.999	0.999	0.000	0.000	0.000	0.000	0.000	0.000	0.000	0.000	0.000	0.000
CB281	2333	M	Forest	0.998	0.998	0.000	0.002	0.000	0.000	0.000	0.000	0.000	0.000	0.000	0.000
CB282	1312	M	Forest	0.953	0.953	0.000	0.016	0.000	0.031	0.000	0.000	0.000	0.000	0.000	0.000
CB42	1115a	M	Forest	0.996	0.996	0.000	0.000	0.001	0.000	0.000	0.000	0.003	0.000	0.000	0.000
CB43	1120	M	Forest	0.995	0.995	0.000	0.003	0.001	0.001	0.000	0.000	0.000	0.000	0.000	0.000
CB45	1122	M	Forest	0.997	0.997	0.000	0.003	0.001	0.000	0.000	0.000	0.000	0.000	0.000	0.000
CB46	1130	M	Forest	0.997	0.997	0.000	0.001	0.001	0.000	0.000	0.000	0.000	0.000	0.000	0.000
CB49	1151	M	Forest	0.992	0.992	0.000	0.003	0.002	0.003	0.000	0.000	0.000	0.000	0.000	0.000
CB50	5012	M	Forest	0.850	0.850	0.000	0.135	0.001	0.012	0.000	0.002	0.000	0.000	0.000	0.000
CB51	1124	M	Forest	0.991	0.991	0.000	0.006	0.002	0.001	0.000	0.000	0.000	0.000	0.000	0.000
CB53	1131	M	Forest	0.999	0.999	0.000	0.000	0.000	0.000	0.000	0.000	0.000	0.000	0.000	0.000
			**Mean**	**0.950**	**0.950**	**0.000**	**0.036**	**0.008**	**0.005**	**0.000**	**0.000**	**0.000**	**0.000**	**0.000**	**0.000**
			**SD**	**0.096**	**0.096**	**0.000**	**0.084**	**0.027**	**0.011**	**0.000**	**0.000**	**0.001**	**0.000**	**0.000**	**0.000**
			**SE**	**0.017**	**0.017**	**0.000**	**0.015**	**0.005**	**0.002**	**0.000**	**0.000**	**0.000**	**0.000**	**0.000**	**0.000**
