# Supplementary material for: Cloacal microbiomes of sympatric and allopatric Sceloporus lizards vary with environment and host relatedness
Source: PLoS One. 2022 Dec 22;17(12):e0279288. doi: 10.1371/journal.pone.0279288 (PMC9779040; doi:10.1371/journal.pone.0279288)
Supplement: S3 File — (PDF) [file pone.0279288.s004.pdf]

M. E. Bunker and S. L. Weiss

Cloacal microbiomes of sympatric and allopatric *Sceloporus* lizards vary with environment and host relatedness

Supporting Information: S3 File. Identities of the 28 ASV's shared among all 3 *Sceloporus occidentalis* populations

PLOS ONE

Shared ASV	Kingdom	Phylum	Family	Class	Order	Genus
ASV_1	Bacteria	Proteobacteria	Gammaproteobacteria	Enterobacteriales	Enterobacteriaceae	Salmonella
ASV_5	Bacteria	Firmicutes	Bacilli	Lactobacillales	Enterococcaceae	Enterococcus
ASV_11	Bacteria	Proteobacteria	Gammaproteobacteria	Enterobacteriales	Enterobacteriaceae	Citrobacter
ASV_12	Bacteria	Proteobacteria	Gammaproteobacteria	Enterobacteriales	Enterobacteriaceae	Hafnia-Obesumbacterium
ASV_18	Bacteria	Cyanobacteria	Oxyphotobacteria	Chloroplast	NA	NA
ASV_32	Bacteria	Bacteroidetes	Bacteroidia	Bacteroidales	Bacteroidaceae	Bacteroides
ASV_35	Bacteria	Firmicutes	Bacilli	Bacillales	Bacillaceae	Bacillus
ASV_37	Bacteria	Bacteroidetes	Bacteroidia	Bacteroidales	Bacteroidaceae	Bacteroides
ASV_61	Bacteria	Bacteroidetes	Bacteroidia	Bacteroidales	Tannerellaceae	Macellibacteroides
ASV_75	Bacteria	Bacteroidetes	Bacteroidia	Bacteroidales	Bacteroidaceae	Bacteroides
ASV_108	Bacteria	Proteobacteria	Deltaproteobacteria	Desulfovibrionales	Desulfovibrionaceae	Bilophila
ASV_116	Bacteria	Bacteroidetes	Bacteroidia	Bacteroidales	Marinifilaceae	Odoribacter
ASV_147	Bacteria	Firmicutes	Negativicutes	Selenomonadales	Veillonellaceae	NA
ASV_180	Bacteria	Bacteroidetes	Bacteroidia	Bacteroidales	Marinifilaceae	Odoribacter
ASV_194	Bacteria	Firmicutes	Clostridia	Clostridiales	Lachnospiraceae	NA
ASV_200	Bacteria	Firmicutes	Clostridia	Clostridiales	Lachnospiraceae	NA
ASV_208	Eukaryota	Parabasalia	Tritrichomonadea	Tritrichomonadea	Monocercomonas	NA
ASV_220	Bacteria	Bacteroidetes	Bacteroidia	Bacteroidales	Marinifilaceae	Odoribacter
ASV_323	Bacteria	Firmicutes	Clostridia	Clostridiales	Lachnospiraceae	NA
ASV_443	Bacteria	Bacteroidetes	Bacteroidia	Bacteroidales	NA	NA
ASV_476	Bacteria	Firmicutes	Clostridia	Clostridiales	Lachnospiraceae	Tyzzerella
ASV_484	Bacteria	Bacteroidetes	Bacteroidia	Bacteroidales	Bacteroidaceae	Bacteroides
ASV_497	Bacteria	Bacteroidetes	Bacteroidia	Bacteroidales	Tannerellaceae	NA
ASV_524	Bacteria	Firmicutes	Clostridia	Clostridiales	Lachnospiraceae	NA
ASV_530	Bacteria	Bacteroidetes	Bacteroidia	Bacteroidales	Rikenellaceae	Alistipes
ASV_995	Bacteria	Verrucomicrobia	Verrucomicrobiae	Verrucomicrobiales	Akkermansiaceae	Akkermansia
ASV_1227	Bacteria	Bacteroidetes	Bacteroidia	Bacteroidales	Rikenellaceae	Millionella
ASV_2049	Bacteria	Proteobacteria	Gammaproteobacteria	Pseudomonadales	Moraxellaceae	Enhydrobacter
